# Exploiting Multimetallic Cooperativity in the Ring-Opening Polymerization of Cyclic Esters and Ethers

**DOI:** 10.1021/acscatal.3c05103

**Published:** 2024-01-08

**Authors:** Utku Yolsal, Peter J. Shaw, Phoebe A. Lowy, Raju Chambenahalli, Jennifer A. Garden

**Affiliations:** School of Chemistry, University of Edinburgh, Joseph Black Building, David Brewster Road, Edinburgh, EH9 3FJ, United Kingdom

**Keywords:** multimetallic cooperativity, heterometallic complexes, ring-opening polymerization, polymerization catalysis, sustainable polymers, polyesters, polyethers

## Abstract

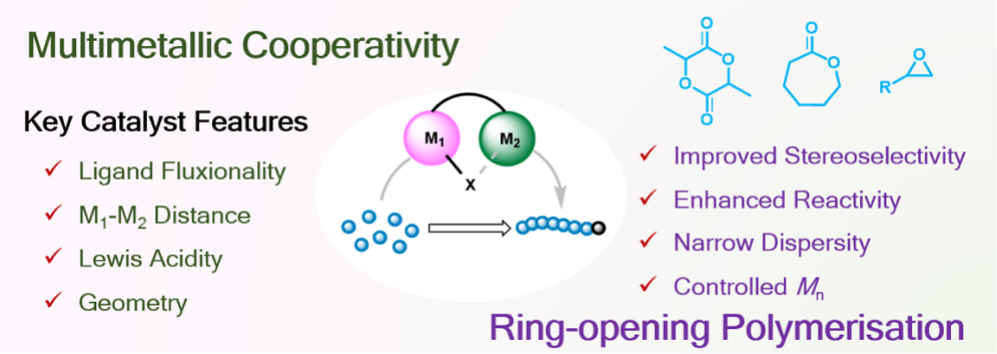

The use of multimetallic complexes is a rapidly advancing route to enhance catalyst performance in the ring-opening polymerization of cyclic esters and ethers. Multimetallic catalysts often outperform their monometallic analogues in terms of reactivity and/or polymerization control, and these improvements are typically attributed to “multimetallic cooperativity”. Yet the origins of multimetallic cooperativity often remain unclear. This review explores the key factors underpinning multimetallic cooperativity, including metal–metal distances, the flexibility, electronics and conformation of the ligand framework, and the coordination environment of the metal centers. Emerging trends are discussed to provide insights into why cooperativity occurs and how to harness cooperativity for the development of highly efficient multimetallic catalysts.

## 1 Introduction

Nature employs bimetallic metalloenzymes as efficient catalysts for a wide variety of chemical transformations, where both metals play an active role in delivering controlled reactivity by positioning the substrates in close proximity.^1^ Inspired by nature, efficient multimetallic catalysts featuring multiple metal centers per complex have been developed for a range of applications including small molecule activation (e.g., C–H bond), asymmetric transformations, and metal–halogen exchange.^2−9^ These complexes are often defined as showing “multimetallic cooperativity”, where the multimetallic species deliver improved catalyst performance compared to their monometallic counterparts.^5,10^ Yet this definition of cooperativity, which is widely used in literature, can be interpreted in different ways and this raises important questions. For example, is it appropriate to compare a multimetallic catalyst to the monometallic analogues, when this often brings differences in the metal coordination environments and the metal concentration? Are the two metals working as a team via cooperative interactions or are both metals acting individually? Does the second metal perform a separate mechanistic role, or does it simply affect the electronic environment and thus the performance of the first metal? While many multimetallic catalysts have been defined as “cooperative”, the origins of such cooperativity often remain vague. This fuels the question, what features make a multimetallic catalyst truly cooperative?

Recent catalyst development has exploited multimetallic and heterometallic cooperativity within homogeneous polymerization catalysts spanning across olefin polymerization, the ring-opening polymerization (ROP) of cyclic esters and ethers, and the ring-opening copolymerization (ROCOP) of epoxides with CO_2_/anhydrides.^11−24^ Within polymer chemistry, cooperative multimetallic catalysts can deliver enhanced catalyst activities and/or improved control over the resultant polymer microstructures. Yet most catalyst design remains focused on monometallic systems. This review focuses on the development of highly efficient multimetallic catalysts for the ROP of cyclic esters and ethers, which are economically and environmentally important processes due to an increasing demand for bioderived and biodegradable oxygenated polymers.^25^ For example, effective organometallic catalysts have been reported for the ROP of renewable monomers such as lactide (LA), ε-caprolactone (ε-CL), and limonene oxide (LO).^26−30^

Within the ROP of cyclic esters and ethers, many multimetallic catalysts have delivered improved polymerization rates as well as controlled polymer structures with targeted number-average molar mass (*M*_n_), narrow dispersity (*Đ*), and high levels of stereocontrol (*P*_r_ or *P*_m_).^16,27,30,31^ But not all multimetallic systems outperform their monometallic analogue(s), and “multimetallic cooperativity” is currently difficult to predict. Understanding the origins of cooperativity and the role of the individual metals is challenging yet highly beneficial for harnessing multimetallic cooperativity. From the studies reported to date, key factors affecting the catalyst performance are emerging including the metal–metal distances (M–M, where M = M or M ≠ M), the coordination geometry and electronic environment of each metal, and the sterics and flexibility of the ligand scaffold.

**Scheme 1:**
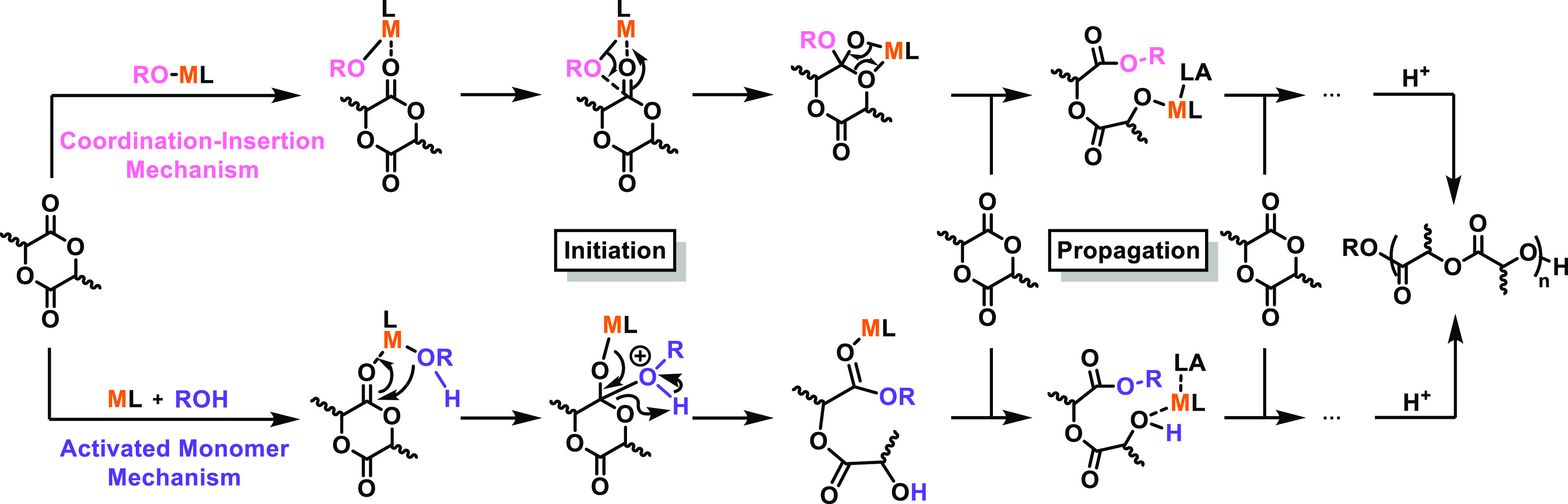
Proposed CIM (Top) and AMM (Bottom) for the ROP of LA Using a Monometallic Catalyst^27,30^

Mechanistic studies have shown that monometallic catalysts for cyclic ester ROP typically proceed via a coordination–insertion mechanism (CIM, Scheme 1, top), where the initiating group is originally part of the catalyst yet becomes incorporated into the polymer chain.^32^ For this reason, organometallic catalysts are often referred to as “initiators”, as the catalyst is not always regenerated into its original form. Other mechanisms, such as an activated monomer mechanism (AMM, Scheme 1, bottom), are also available.^27,30^ For both mechanisms, the metal acts as a Lewis acid to coordinate and activate the monomer toward nucleophilic attack. However, in CIM, the nucleophile is typically a metal-alkoxide, alkyl, amido, or halide group, whereas for AMM, an exogeneous nucleophile such as an alcohol is used. With an AMM, the metal complex is unchanged and thus is a true catalyst. As some organometallic complexes can operate through a combination of CIM and AMM,^27,30^ here we use the term “catalyst” in all cases.

**Figure 1:**
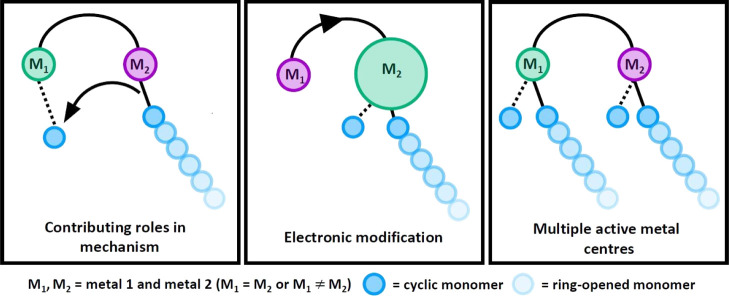
Illustration of potential roles of different metals in bimetallic systems.

A greater number of potential ROP mechanisms are available with multimetallic catalysts, as the functions of each metal can vary. For example, in some cooperative multimetallic ROP catalysts, each metal catalyzes different steps, with one metal providing monomer coordination sites (Figure 1, left, M_1_) while the other acts as the source of the nucleophile (Figure 1, left, M_2_).^33−35^ The electronics of the first metal center can also be modified by incorporating a second metal, providing an alternative method of fine-tuning the metal Lewis acidity compared to the usual route of altering the ligand substituents, which can involve time-consuming and synthetically challenging ligand modifications (Figure 1, center).^36^ Or, multimetallic systems can display improved reactivity simply because multiple active metal centers are operating simultaneously (Figure 1, right). Metals working individually but both contributing to the polymerization can be affected by the proximity of the second metal as well as steric hindrance, either from the ligand scaffold or from a growing polymer chain. More than one of these modes of activity enhancement may occur simultaneously.^33,34^ While a variety of multimetallic ROP mechanisms have been proposed, which bear some similar features to the monometallic mechanisms (monomer coordination and nucleophilic attack), a well-defined overarching multimetallic mechanism has not yet been identified.^31,36^

Understanding the key features of efficient multimetallic catalysts is crucial to harnessing metal–metal cooperativity and improving catalyst performance. Indeed, the ring-opening polymerization of cyclic esters and epoxides has been the focus of several review articles,^32,37−42^ including those focusing on bi- and multimetallic catalysts.^31,43^ Therefore, rather than providing a comprehensive overview of the field, this review highlights catalysts for the homopolymerization of ε-CL, LA, and epoxides that have delivered insight into multimetallic cooperativity, to investigate the potential origins of cooperativity and identify patterns and trends.

## 2 Multimetallic Catalysts for the ROP of Cyclic Esters

### 2.1 Homobimetallic Catalysts

#### 2.1.1 Enhanced Polymerization Activity

**Figure 2:**
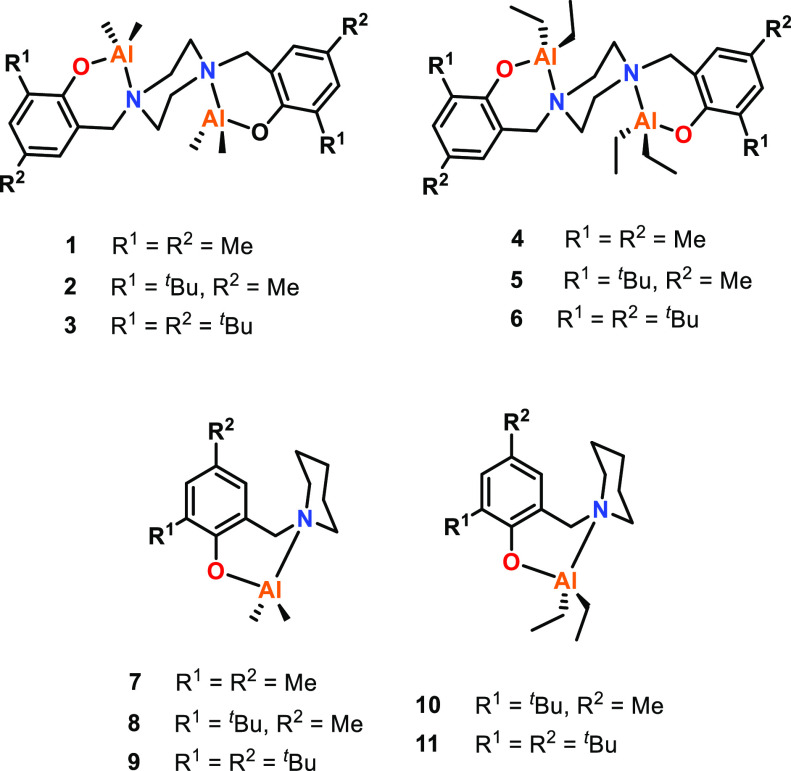
Mono- and bimetallic phenolato-Al complexes. Tested for ε-CL ROP, where bimetallic complexes showed 2–8 times higher activities than those of their monometallic counterparts.^50^

Cyclic ester ROP has been used as a method of producing aliphatic polyesters for decades, and monometallic tin(II) octoate is currently the industrial catalyst of choice for polylactic acid (PLA) production. More recently, multimetallic catalysts have emerged as highly efficient initiators for lactide ROP as well as ε-caprolactone ROP to produce polycaprolactone (PCL).^44−49^ The term “multimetallic cooperativity” is most often applied to multimetallic catalysts with higher activities than the monometallic analogue(s), and most of the reported examples are bimetallic complexes. For example, Yuan, Yao, and co-workers synthesized and tested various mono- and bimetallic Al complexes for ε-CL ROP (**1**–**11**, Figure 2),^50^ where the bimetallic complexes exhibited 2–8 times higher activity than that of the monometallic analogues. Specifically, bimetallic **5** gave a *k*_obs_ value of 1.28 × 10^–4^ s^–1^, compared to 3.38 × 10^–5^ s^–1^ for monometallic **10** (70 °C, toluene, with [ε-CL]:[catalyst (Cat)] loadings of 400:1 for bimetallic **5** and 200:1 for monometallic **10**). As both systems had similar steric environments, the difference in rates was attributed to cooperative interactions between the two Al centers in **5**. The Gibbs energy of activations were also calculated, and the energy barrier for ROP initiation was 2 kcal mol^–1^ lower for **5** than for **10**.

**Figure 3:**
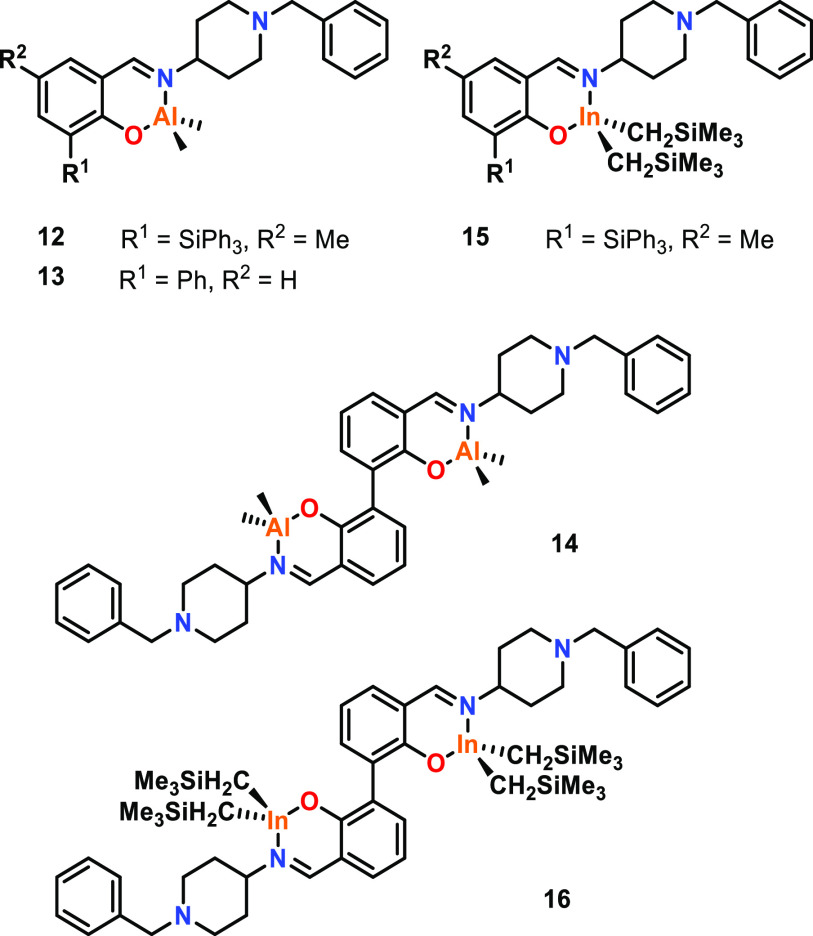
Mono- and bimetallic Al and In complexes based on 2,2′-bisphenolate ligands. Tested for LA ROP, in which Al dimers showed enhanced polymerization over monometallic counterparts, whereas In did not.^51^

In 2013, Carpentier, Kirillov, and co-workers screened a range of mono- and bimetallic complexes for the ROP of racemic lactide (*rac*-LA), using 2-propanol (^*i*^PrOH) as an initiator.^51^ In general, bimetallic Al complex **14** gave catalyst activities 5–10 times higher than those of monometallic **12** and **13** (Figure 3). For example, the apparent rate constant for **14** was reported as 12.2 × 10^–3^ s^–1^ at 110 °C, compared to 1.1 × 10^–3^ s^–1^ and 2.5 × 10^–3^ s^–1^ for **12** and **13**, respectively (toluene, with a [*rac*-LA]:[Cat]:[^*i*^PrOH] ratio of 1000:1:10 for bimetallic **14** and 500:1:5 for monometallic **12** and **13**). Eyring analyses on the kinetic data from **14** and **12** showed that the free energy barrier for bimetallic **14** was roughly 2 kcal mol^–1^ lower in energy than that of monometallic **12**. The enhanced polymerization activity of **14** was attributed to potential cooperative effects between the two active centers. While single crystal X-ray diffraction (XRD) studies of **14** showed an M–M distance of 8.0 Å, rotation about the aryl–aryl bond may bring the metal centers into closer proximity in solution, facilitating cooperation (refer to section 2.1.2 for further detail on the importance of M–M distances).^31^ The same ligand frameworks were also used with indium, yet no significant activity differences were observed between the monometallic and bimetallic systems (**15** and **16**). This difference between the Al and In systems was attributed to the potentially different polymerization mechanisms with indium (AMM) and aluminum (CIM), as the In-alkyl center did not react with ^*i*^PrOH to form the In-alkoxide under the polymerization conditions. These results highlight that multimetallic cooperativity can depend on the choice of metal and the polymerization mechanism.

**Figure 4:**
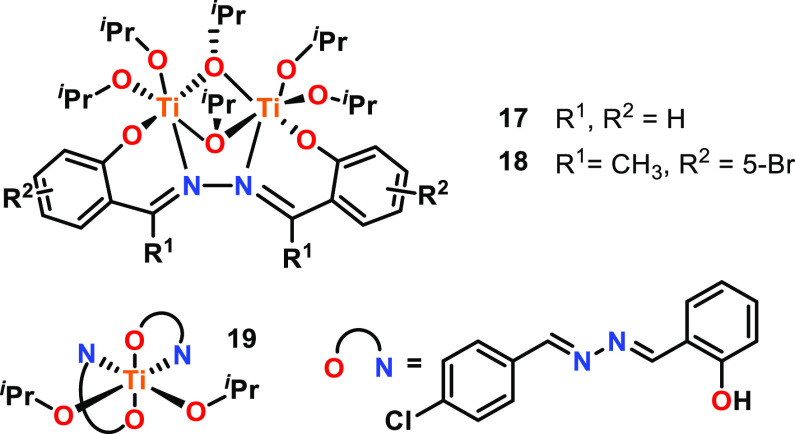
Representative mono- and bimetallic titanium complexes using hydrazine-bridged Schiff base ligands. Tested for l-LA ROP where bimetallic complexes showed 10–60 times greater activity than that of monometallic **19**.^52^

Polymerization rate enhancements have also been observed with a series of bimetallic Ti complexes featuring hydrazine-bridged Schiff base ligands for the ROP of l-LA (refer to **17** and **18**, Figure 4 for representative examples).^52^ All bimetallic complexes exhibited 10–60 times higher catalytic activities compared to monometallic **19** (60 °C, toluene; for bimetallic complexes, [l-LA]:[Cat] 100:1, for monometallic complexes, [l-LA]:[Cat], 100:2). For example, bimetallic **17** and **18** gave respective *k*_obs_ values of 0.065 min^–1^ and 0.014 min^–1^, whereas monometallic **19** was particularly slow with a *k*_obs_ value of 0.001 min^–1^. Kinetic and spectroscopic studies together with the polymer characterization data suggests that the bimetallic Ti complexes remain intact during the polymerization, with initiation only occurring from the terminal isopropoxide groups.

#### 2.1.2 Cooperative Interactions and Metal–Metal Distances

**Figure 5:**
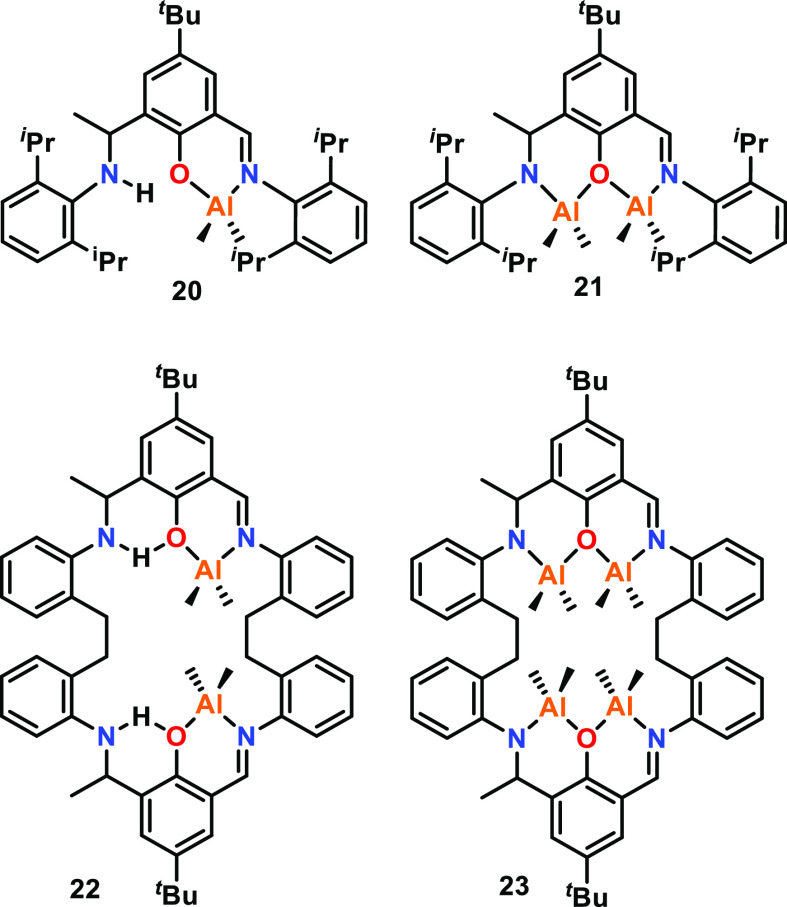
Schiff base ligand-derived Al complexes. Tested for ε-CL ROP where bimetallic **22** exhibited greater activity than that of tetrametallic **23**, and monometallic **20** exhibited greater activity than that of bimetallic **21**. Complexes **20** and **21** with the open ligand framework were less active than that of **22** and **23** using a cyclic ligand.^53^

While the origins of metal–metal cooperativity are not always clear, the metal–metal distance has emerged as a key factor.^31,43^ Where possible, metal–metal distances for the solid-state structures have been obtained from XRD data, although it is important to note that these values provide a somewhat limited comparison for the solution-state structures present under polymerization conditions. Redshaw and co-workers investigated the influence of M–M distances using macrocyclic Schiff base alkylaluminum complexes for the ROP of ε-CL (Figure 5), and showed that catalysts **20**–**23** were all active but exhibited significantly different reactivities (25 °C, toluene, [ε-CL]:[Al]:[BnOH] 500:1:1, where BnOH is benzyl alcohol).^53^ Notably, these catalysts are unusual examples of asymmetric ligand frameworks that encapsulate multiple metals in close proximity. In particular, tetrametallic **23** was less active than bimetallic **22**, which was attributed to the closer Al–Al distances in **23** (3.21 and 3.23 Å vs 5.78 Å in **22**). This study was one of the first to suggest there may be an optimal M–M distance for ε-CL ROP catalyzed by multimetallic complexes. The longer M–M distance in **22** may enable coordination of a single ε-CL monomer, where one metal center acts as a Lewis acid and the second provides the initiating group or propagating chain to ring-open the coordinated monomer (refer to section 2.1.4 for further mechanistic details). Following a similar trend, bimetallic **21** was less active than monometallic **20**, and both were less active than **22** and **23**, suggesting that cooperative interactions may be hindered if the metals are too close and/or feature an aluminoxane Al–O–Al unit.

**Figure 6:**
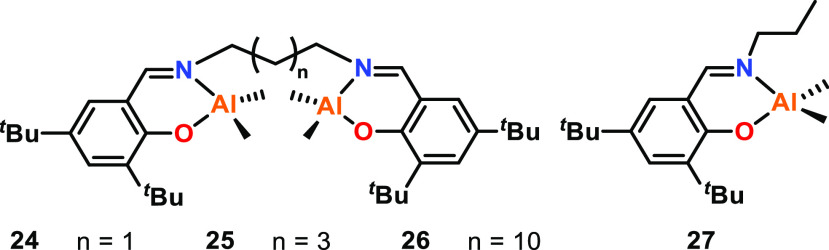
Mono- and bimetallic salen aluminum complexes. Tested for *rac*-LA ROP, the enhanced activity of **24** was attributed to the short alkyl bridge whereas complexes **25** and **26** exhibited similar activity to **27**, indicating the metal centers at greater distances were acting independently.^35^

While the M–M distances can be “too short”, they can also be “too long”. Mazzeo and co-workers prepared three bimetallic salen aluminum complexes for the ROP of *rac*-LA or cyclohexene oxide (CHO), with varying Al–Al distances (**24**–**26**, Figure 6).^35^ Complex **24**, with the shortest alkyl bridge, gave the highest activity toward *rac*-LA with a turnover frequency (TOF) of 16.7 h^–1^ compared to 3.5 h^–1^ and 3.1 h^–1^ for **25** and **26**, respectively (70 °C, toluene, [*rac*-LA]:[Cat]:[^*i*^PrOH], 200:1:4). While the Al–Al distances were not quantified for these complexes, the enhanced activity of **24** was attributed to the short Al–Al distance potentially disfavoring two separate polymer chains per catalyst due to steric hindrance, and instead favoring multimetallic cooperativity. With **25** and **26**, the metal centers were proposed to act independently due to the longer Al–Al distances, as monometallic **27** gave comparable activity to **25** and **26** for *rac*-LA ROP under the same conditions. Both NMR spectroscopic and kinetic studies suggested that **24** reacts with ^*i*^PrOH to form the Al-alkoxide significantly faster than **25** and **26**; no induction period was observed for **24**. Therefore, the metal–metal proximity is also likely to play a role in the activation of the catalyst.

**Figure 7:**
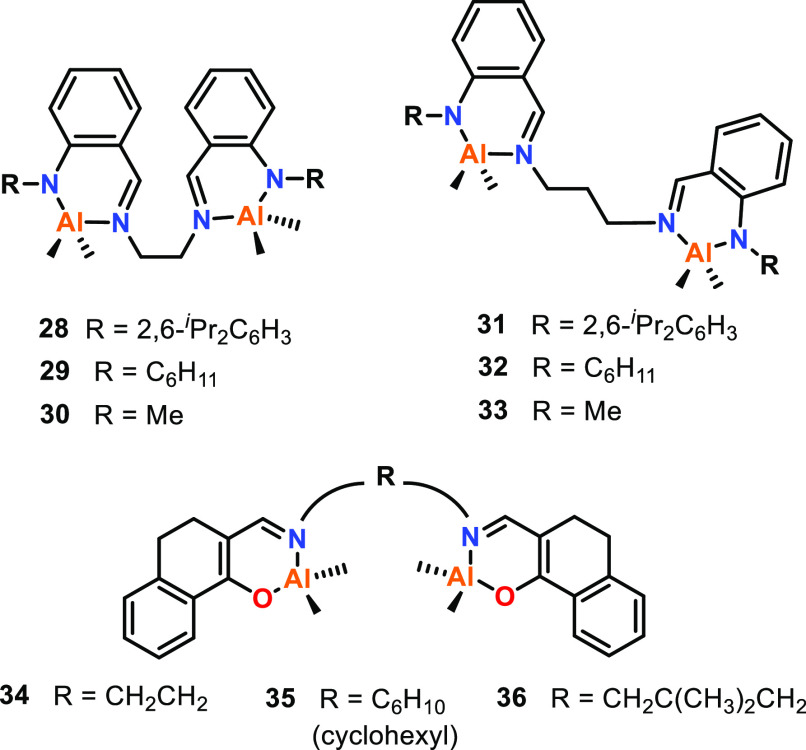
Various bimetallic aluminum complexes prepared using spacer bridging groups. Shorter ethyl-bridged complexes **28**–**30** gave higher activities than those of propyl-bridged complexes **31**-**33** for *rac*-LA ROP. In contrast, complexes **34**–**36** showed a correlation between increased M–M proximity and increased activity in ε-CL ROP (**36** > **34** > **35**).^24,54^

Multimetallic complexes using spacer bridging units have also been reported by others including Shaver (**28**–**33**, Figure 7) and Li (**34**–**36**).^24,54^ Complexes **28**–**33** were screened for the ROP of *rac*-LA, with the ethyl bridged catalysts (**28**–**30**) giving higher polymerization rates than those of the propyl bridged catalysts (**31**–**33**).^24^ Complexes **34**–**36** exhibited fast activity for ε-CL ROP (80 °C, toluene, [ε-CL]:[Cat]:[^*i*^PrOH], 100:1:1), in the order **36** (TOF; 1660 h^–1^) > **34** (582 h^–1^) > **35** (384 h^–1^). An increase in reactivity correlates with the increase in Al–Al distances observed in the XRD data, where **36** had the longest Al–Al distance (7.77 Å), **35** had the shortest (5.97 Å), and **34** was intermediate (6.62 Å). The flexible structure of **36** was proposed to allow the metal centers to approach each other in solution and improve cooperativity. Notably, this correlation differs from some of the aforementioned multimetallic Al catalysts, where a shorter Al–Al distance gives enhanced activity, indicating that spacer sterics and ligand flexibility may also influence multimetallic cooperativity.

**Figure 8:**
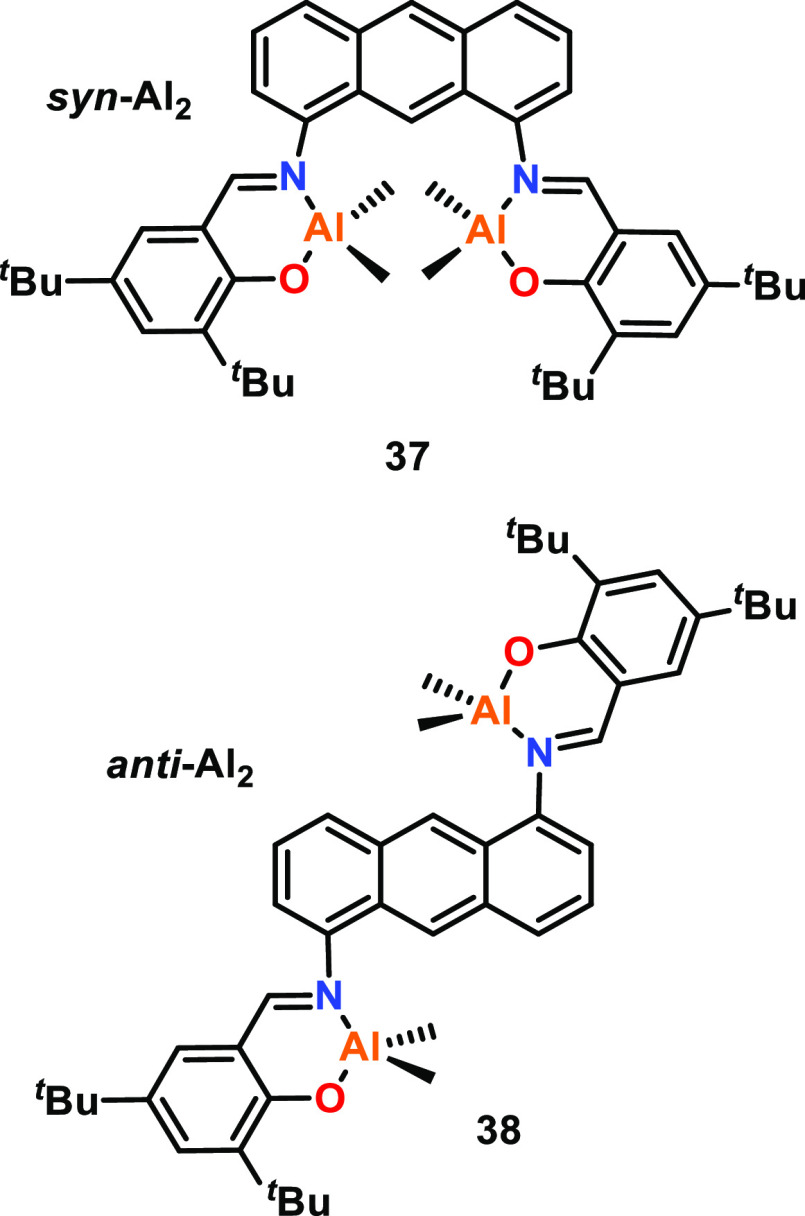
Bimetallic aluminum complexes supported by *syn*- and *anti*-bis(salicylaldimine) ligands; *syn*-**37** showed greater activity than that of *anti*-**38** for *rac*-LA ROP.^55^

Liu, Li, and co-workers investigated the effect of M–M distances using two bimetallic Al complexes supported by bis(salicylaldimine) ligands, featuring a rigid anthracene skeleton with “syn” and “anti” conformations (**37** and **38**, respectively, Figure 8).^55^ Single crystal XRD characterization of *syn*-**37** revealed an Al–Al distance of 6.67 Å, which was predicted to be significantly shorter than the M–M distance in *anti*-**38**. While suitable crystals of **38** could not be grown, XRD analysis of the ligand framework provided support for the M–M distance in *anti*-**38** being significantly longer than *syn*-**37**, as the O–O distances were determined as 10.64 and 4.23 Å in the *anti*-bis(salicylaldimine) and *syn*-bis(salicylaldimine) ligands, respectively. While both catalysts were efficient for *rac*-LA ROP with 1 equiv of benzyl alcohol (BnOH), *syn*-**37** gave higher catalytic activities than *anti*-**38**, with respective *rac*-LA conversions of 94% and 85% under identical conditions (70 °C, toluene, [*rac*-LA]:[Cat]:[BnOH], 100:0.5:1). Both **37** and **38** contain two methyl groups per aluminum center, which can be replaced by alkoxide groups, and the stabilities of these two catalysts were tested against an increased number of equivalents of BnOH. While the catalysts were active in the presence of 2 equiv of BnOH, **38** started to degrade with 4 equiv of BnOH. Further studies revealed that excess BnOH (10 equiv) can degrade both complexes by reforming the pro-ligands, but **37** was more tolerant to alcohols and the decomposition was slower. These differences in the catalyst stabilities were proposed to be due to cooperative interactions between the two metal centers or steric hindrance arising from two Al centers in close proximity.

**Figure 9:**
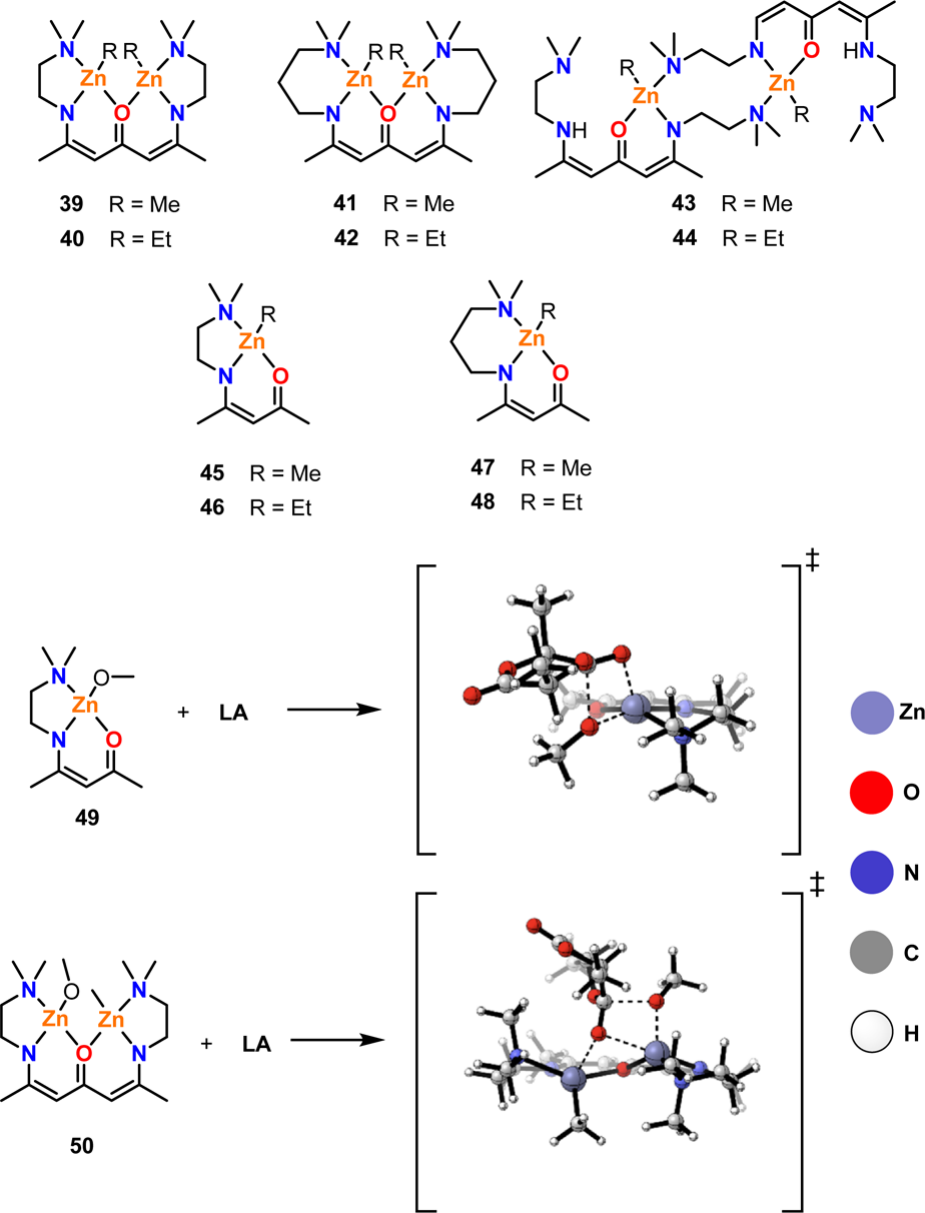
Bimetallic ketodiiminate zinc-alkyl complexes investigated for the ROP of l-LA, along with the monometallic analogues, where complexes **39**–**42** outperformed **43** and **44**, and monometallic **45**–**48** gave lower activities than those of the bimetallic counterparts.^56^ Model complexes **49** and **50** were used for quantum mechanical calculations. In the drawn transition states, slate blue: zinc; red: oxygen; blue: nitrogen; gray: carbon; white: hydrogen. Adapted from Ghosh, S.; Schulte, Y.; Wölper, C.; Tjaberings, A.; Gröschel, A. H.; Haberhauer, G.; Schulz, S. Cooperative Effect in Binuclear Zinc Catalysts in the ROP of Lactide. *Organometallics***2022**, *41* (19), 2698–2708. Copyright 2022 American Chemical Society.

The effects of M–M distances have also been investigated in bimetallic zinc complexes. Recently, Schulz and co-workers reported the syntheses of ketodiiminate zinc-alkyl complexes (Figure 9) and studied their activity for the ROP of l-LA.^56^ In general, bimetallic complexes **39**–**44** were highly active with the Zn-ethyl complexes giving enhanced TOF values compared to the Zn-methyl analogues, likely due to the higher nucleophilicity of the ethyl groups. The complexes were characterized using single crystal XRD, and the Zn–Zn distances were determined to be 3.33, 3.33, 3.43, 5.05, and 5.03 Å, for **39**, **41**, **42**, **43**, and **44**, respectively. Therefore, two main M–M distances were compared in this study, i.e. ∼3 Å vs ∼5 Å. Diffusion-ordered NMR spectroscopy (DOSY NMR) was also used to confirm that the complexes adopt similar aggregation states in both the solid and solution state. Complexes **39**–**42** (∼3 Å) were significantly more active than **43** and **44** (∼5 Å), with the TOF values of **39**–**42** ranging from 177 to 388 h^–1^ compared to 76 h^–1^ for **43** and 129 h^–1^ for **44** (room temperature, RT, [l-LA]:[Cat] 200:1). In addition, the relationship between the observed and theoretical molar mass values indicated two chains growing from **43** and **44** yet only a single chain growing for the ∼3 Å catalysts. These results suggest that the two Zn centers in **39**–**42** work cooperatively and indicate that a distance of ∼5 Å is too far for metal–metal cooperativity in this case. Kinetic studies also revealed that both the initiation and propagation were faster with **39** and **40** compared to **43** and **44**. The monometallic zinc analogues **45**–**48** were investigated under identical conditions and gave lower TOF values, ranging from 26 to 86 h^–1^ (RT, [l-LA]:[Cat] 200:1). Quantum chemical calculations were performed on model complexes **49** and **50**, where OMe was modeled as a simplified version of the propagating polymer chain (Figure 9). These studies revealed that the transfer of the methoxy group to l-LA has a lower energy barrier for **50** compared to **49**, as the second Zn center improves the activation of the carbonyl bond, resulting in faster polymerization.

The studies highlighted in this section emphasize the importance of M–M distances in establishing cooperativity between metal centers, with a “Goldilocks” scenario required for the most efficient catalysis. If the M–M distance is too long, it can result in the two metal centers acting separately, without cooperation. Similarly, the M–M distance can be too short, potentially due to steric hindrance, preventing effective polymerization. These initial reports indicate that the optimal M–M distance may vary for different metals and different ligand systems, and more research would help to provide further understanding to identify overall trends. This includes the investigation of tri- and tetrametallic systems, some examples of which have already been reported (refer to section 2.2).

#### 2.1.3 Effects of Ligand Flexibility, Electronics and Conformations on Metal–Metal Cooperativity

**Figure 10:**
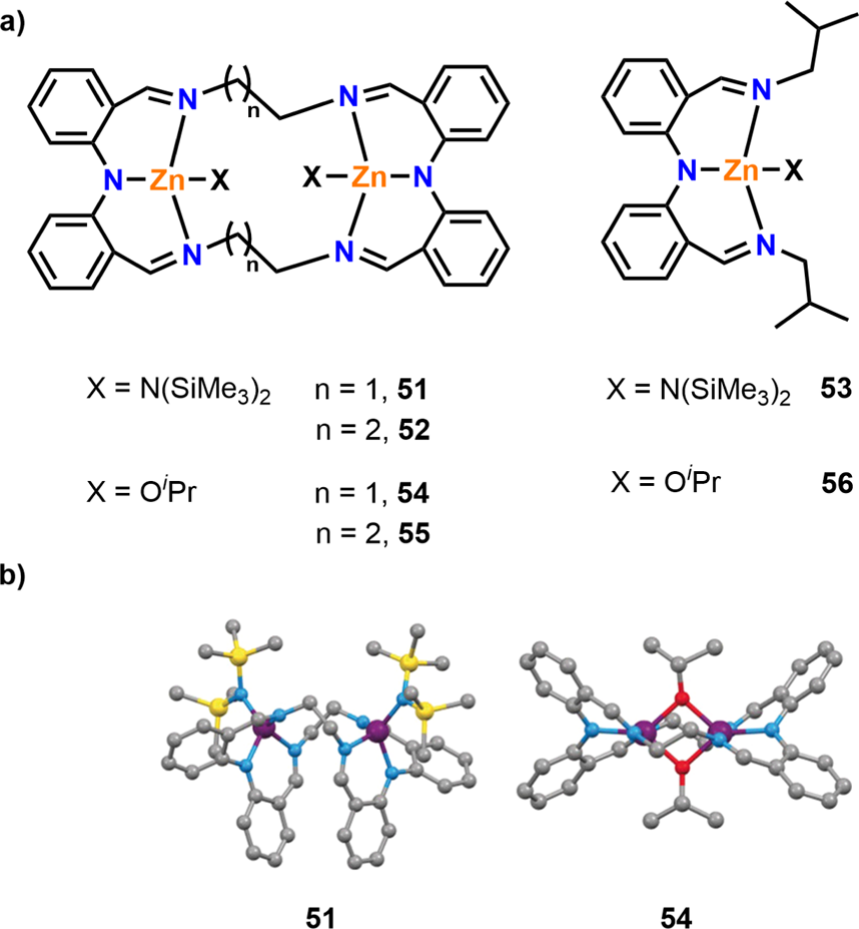
(a) Zinc complexes for the ROP of *rac*-LA. (b) The obtained XRD molecular structures for **51** and **54** (blue: nitrogen, red: oxygen; purple: zinc; gray: carbon; yellow: silicon).^58^ Bimetallic amido complexes **51** and **52** demonstrated multimetallic cooperativity compared to monometallic **53** for *rac*-LA ROP, whereas monometallic **56** displayed similar activity to bimetallic **55** and outperformed **54**. Adapted with permission from ref (58). Copyright 2016 John Wiley and Sons.

While the M–M distance is key, this is generally underpinned by the structure of the ligand, which also influences the electronics at the active metal center. Flexible ligands have long been known to improve the performance of monometallic aluminum salen catalysts,^57^ which is generally attributed to the ligand flexibility facilitating access to key transition states. With multimetallic catalysts, the ligand also plays a key role and can either enhance or disfavor cooperativity. For example, Brooker, Williams, and co-workers reported a key study highlighting the importance of ligand conformation in *rac*-LA ROP, which investigated mono- and dizinc catalysts with amido and alkoxide initiating groups (Figure 10).^58^ Bimetallic amido complexes **51** and **52** exhibited remarkably high activities for the ROP of *rac*-LA (RT, THF, [*rac*-LA]:[Cat] 1000:1), giving respective TOFs of 20 300 h^–1^ and 45 000 h^–1^, and polymerized 1000 equiv of monomer within 1 min in the absence of exogenous alcohols. The activity of monometallic **53** was determined as 14300 h^–1^ under the same conditions, which was three times slower than bimetallic **52**, suggesting multimetallic cooperativity between the active Zn centers. The alkoxide analogues (**54**–**56**) were also active and gave improved control over the polymer structure, albeit with lower TOF values than that of the amido analogues. To understand the difference in polymerization rates between **51** and **54**, the structures were determined using XRD. Complex **51** exists in a folded confirmation, whereas **54** adopts a planar conformation due to the alkoxide groups bridging between two metal centers (Figure 10b). The folded conformation was proposed to enable a strong electron donation from the ligand system with short M–M distances and available coordination sites on Zn, and TOF values as high as 60 000 h^–1^ were obtained using **51** under optimized (immortal) conditions.

**Figure 11:**
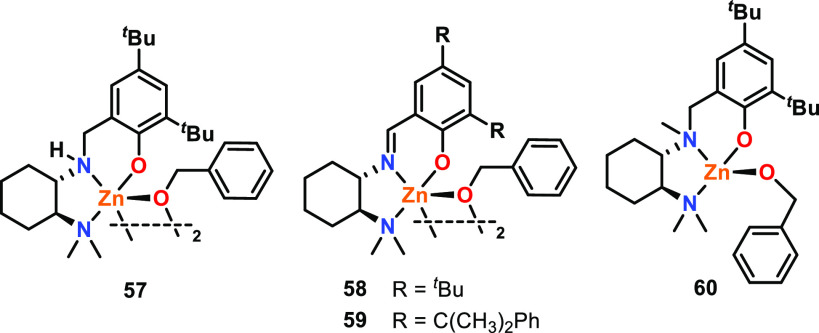
Diamino- and aminoimino phenolate-supported zinc-benzoxide complexes. Tested for *rac*-LA ROP where bimetallic complexes **57**–**59** outperform monometallic **60**.^59^

The role of amine or imine donors in various Zn complexes was investigated by Mehrkhodavandi and co-workers (Figure 11), by varying the nature of the central nitrogen donor from a secondary amine (**57**) to an imine (**58** and **59**) and a tertiary amine (**60**).^59^ XRD studies combined with pulsed field-gradient spin echo ^1^H NMR studies showed that while **57**–**59** were bimetallic, **60** was monometallic. Complexes **57**–**59** also significantly outperformed **60** in *rac*-LA ROP, reaching full conversions of *rac*-LA in less than 10 min even with a 1000:1 loading of [*rac*-LA]:[Zn], whereas **60** took 4 h to reach full conversion under the same conditions (25 °C, CH_2_Cl_2_). The polymers obtained using **58** and **59** also showed good stereocontrol, with *P*_r_ values of 0.80 and 0.68, respectively, whereas **60** yielded atactic PLA. As the dimethylamino group is a labile Lewis donor, the ligands of **57**–**60** have the potential to act as κ^2^- or κ^3^-coordinating ligands (ON or ONN, respectively). The XRD studies showed that in the solid state, the ligands of **57**–**59** are κ^2^-coordinated, with tetracoordinate Zn bonding to the κ^2^-ligand and two bridging OBn groups to form a bimetallic structure. In contrast, the ligand is κ^3^-coordinated in **60**, and so the structure is monometallic. These observations indicate that subtle changes in the ligand design and the ligand lability can facilitate or hamper multimetallic cooperativity.

**Figure 12:**
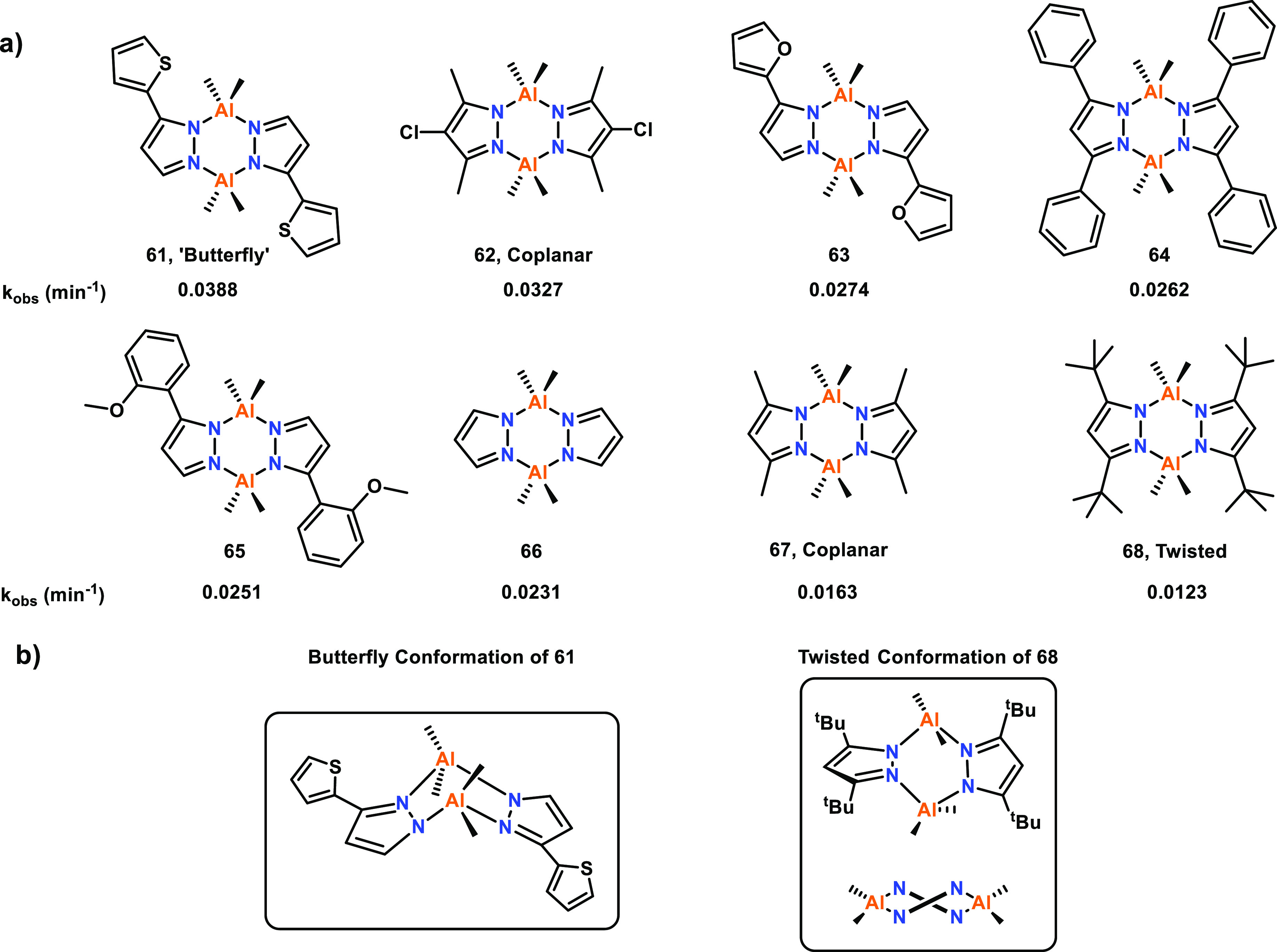
(a) Bimetallic Al complexes bridged with symmetrical and asymmetrical pyrazole ligands with different substituents. (b) The butterfly and twisted conformations of **61** and **68**. Tested for ε-CL ROP with catalyst activity decreasing in order from **61** to **68**.^34^

The effects of ligand conformation have also been investigated for bimetallic Al complexes, including those based on symmetric and asymmetric pyrazole ligands that were tested for the ROP of ε-CL (**61**–**68**, Figure 12).^34^ XRD data revealed Al–Al distances of 3.60, 3.78, 3.77, and 3.53 Å for **61**, **62**, **67**, and **68**, respectively. While molecular structures were only reported for four of the eight complexes, the reported metal–metal distances are within 0.3 Å of each other, and thus the other complexes could be expected to give similar Al–Al distances. Due to the similarity in the M–M distances, the different catalytic activities could be mostly attributed to the electronic effects from electron-donating or -withdrawing ligand substituents and the different conformations adopted by the ligand framework.

The catalyst activity decreased in order from **61** (most active) to **68** (refer to Figure 12 for *k*_obs_ values, RT, toluene, [ε-CL]:[Cat]:[BnOH] 100:0.5:2). Notably, catalysts **67** and **68** also showed substantial induction periods of 113 and 39 min, respectively. Ligand frameworks with electron-withdrawing substituents, such as **62**, generally enhanced the polymerization rate, whereas electron-donating groups decreased the catalyst activity (e.g., catalyst **68**); this may be because the electron-withdrawing substituents enhance the Lewis acidity of Al. The ligand conformation can also impart strong effects on the polymerization rate, potentially by affecting the sterics around the metal centers. For example, **61** shows a “bent butterfly” conformation and gives the best performance of all the complexes; this conformation gives **61** more available space for monomer coordination than a flat Al_2_N_4_ arrangement (Figure 12b, left). A cooperative polymerization mechanism was proposed for **61**, which is further discussed in section 2.1.4. In contrast, **68** gave the poorest catalyst performance, which was attributed to the twisted confirmation decreasing the available space for ε-CL coordination as the metal centers become more hindered (Figure 12b, right).

**Figure 13:**
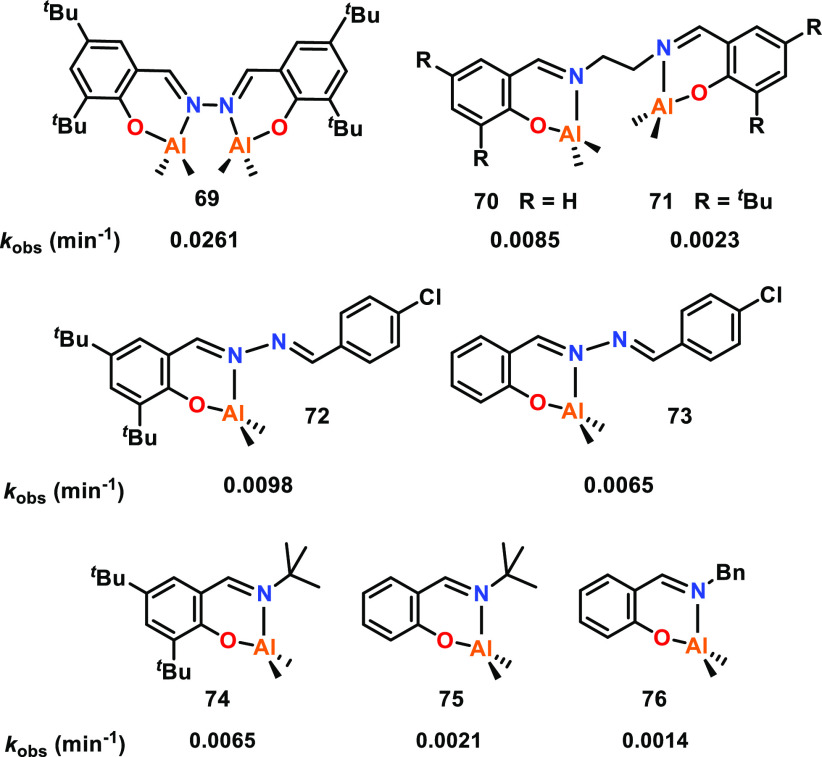
Comparative catalytic activities of various mono- and bimetallic Al complexes. Tested for ε-CL ROP with bimetallic **69** displaying the highest catalyst activity.^60^

A series of hydrazine-bridging Schiff base and salen Al complexes were synthesized and studied for the ROP of ε-CL, to compare the activities of monometallic and bimetallic complexes (**69**–**76**, Figure 13).^60^ Complexes **69**–**76** were all active for the polymerization of ε-CL (RT, toluene, for bimetallic complexes [ε-CL]:[Cat]:[BnOH] 100:0.5:2; for monometallic complexes [ε-CL]:[Cat]:[BnOH] 100:1:2). The catalytic activity of bimetallic hydrazine-bridged **69** was higher than all of the other Al complexes investigated in this study, and 3 to 11-fold higher than the salen aluminum complexes. Interestingly, the less sterically hindered **70** was *ca.* four times more active than **71**. While this suggests that bulky ligand substituents can decrease the catalytic activity, an opposite trend was observed with the monometallic Al complexes where increased steric bulk correlated with improved catalyst activity (e.g., **74** vs **75**). The hydrazine bridging systems (**69** vs **70**, **72** and **73** vs **74**–**76**) exhibited higher activities, which may arise from the electron-withdrawing hydrazine group enhancing the Lewis acidity of Al, thus increasing the polymerization rate. The enhanced activities of bimetallic Al hydrazine-bridged **69** vs monometallic hydrazine complexes **72** and **73** mirrors the trend observed for related Ti hydrazine-bridged complexes, where the bimetallic complexes also outperformed the monometallic analogues (see **17** and **18**, Figure 4, section 2.1.1, for representative examples).^52^

Overall, the studies in this section show that the ligand flexibility, lability, sterics, and electronics can significantly impact the performance of bimetallic catalysts in ROP. Certain ligand conformations can improve polymerization rates, either by bringing metal centers into closer proximity to each other or by providing more space for monomer coordination.^34,58^ For catalysts following a CIM, the monomer coordination and metal-alkoxide release are key mechanistic steps, and the ligand sterics and electronics have a direct impact on both. Fine-tuning the metal accessibility as well as the Lewis acidity and M–M proximity can help to access multimetallic cooperativity and boost the catalyst performance.

#### 2.1.4 Mechanistic and Electronic Origins of Cooperativity in Multimetallic ROP Catalysis

Monometallic catalysts for the ROP of cyclic esters typically operate via a coordination–insertion and/or an activated monomer mechanism (refer to Introduction, Scheme 1), where the mechanistic pathway depends on the catalyst structure and nucleophilicity. For example, a highly reactive metal–alkoxide bond typically favors the CIM, while an unreactive metal–alkoxide bond facilitates the AMM.^61,62^ In contrast, the mechanism for multimetallic catalysts is not yet well understood, and different ROP mechanisms have been proposed for different multimetallic catalysts. Here, we aim to bring together and discuss some of the proposed mechanisms to identify some of the key hypotheses and trends.

**Scheme 2:**
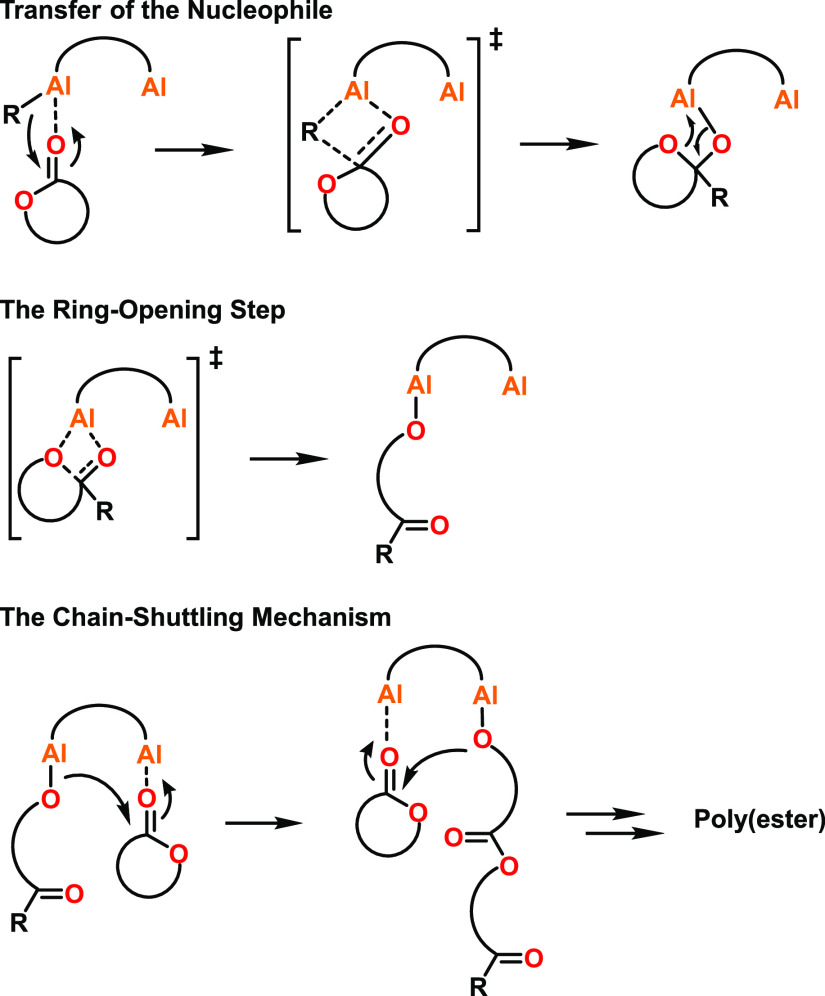
Proposed Initiation and Chain Shuttling Mechanism for Homopolymerization of l-LA and ε-CL Using Some Bimetallic Al Catalysts^35,50^

Most mechanisms proposed for multimetallic ROP catalysts rely on coordination–insertion pathways that have been adapted to include the participation of the other metal center(s).^35,50,56^ For example, Yuan, Yao, and co-workers proposed a “chain shuttling” mechanism for the ROP of ε-CL with bimetallic alkyl Al catalysts (**1**–**6**, Figure 2), where ε-CL coordinates to one of the Al centers and is ring-opened by the Al-alkyl group (Scheme 2).^50^ Subsequent coordination of another ε-CL to the neighboring Al center enables ring-opening by the propagating chain end. The polymerization was proposed to continue by ε-CL coordination to the vacant Al site, with the propagating chain “shuttling” between the metal centers upon ring-opening.

**Scheme 3:**
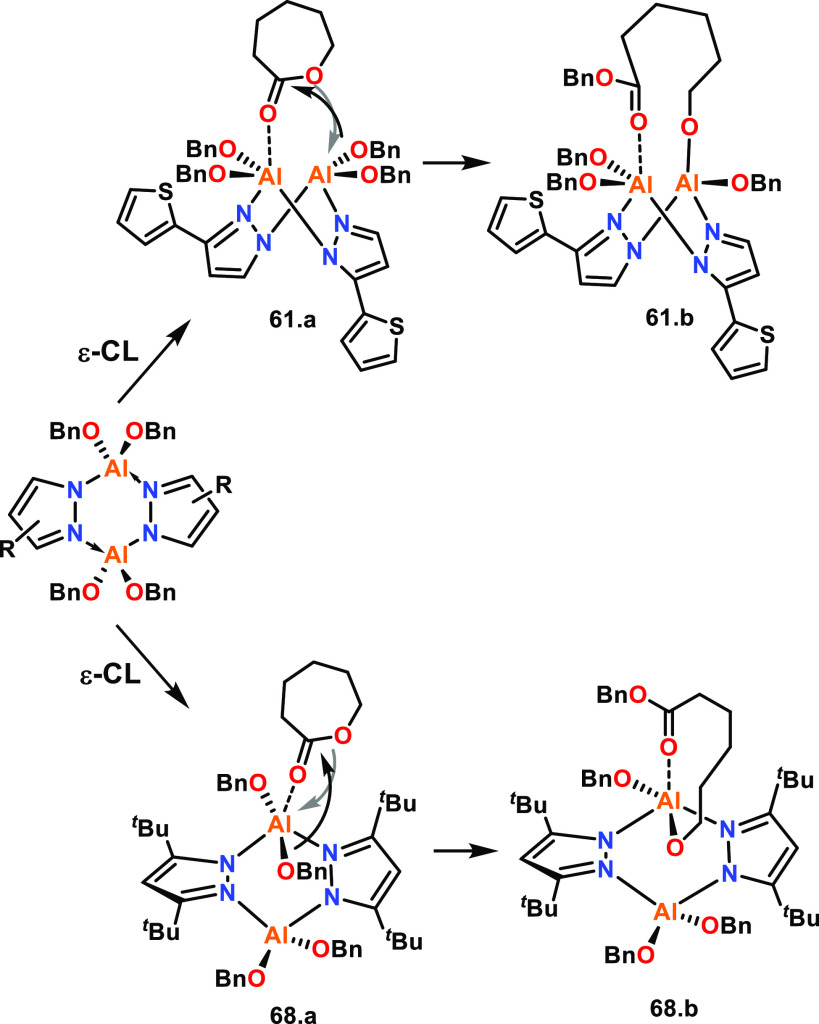
Plausible Mechanisms for the ROP of ε-CL Using Bimetallic Al Pyrazole Complexes^34^

A similar chain-shuttling mechanism was proposed by Mazzeo and co-workers for the ROP of l-LA using bimetallic Al salen catalysts (**24**–**26**, Figure 6).^35^ This mechanism also involved cyclic ester monomer coordination to one of the Al centers but differs in that the initiating group comes from the neighboring Al (an alkoxide, compared to the previous system where an alkyl initiator was used). Other studies have suggested that the ligand framework can influence the transfer of the nucleophile,^34^ with the sterics around the metal centers affecting whether the initiating group comes from the monomer-coordinated metal center or the vacant metal center in bimetallic Al-based systems. For example, complexes **61** and **68** (refer to Figure 12, section 2.1.3) were activated using BnOH and reacted with ε-CL, which was proposed to form the respective species shown in Scheme 3 (**61.a/b** and **68.a/b**). In this study, the bent butterfly conformation of **61** was proposed to provide more space around the active centers, enabling the other Al center to provide the alkoxide in a cooperative manner. With **68**, the twisted ligand conformation was proposed to give less space around the active centers, disfavoring metal–metal cooperativity, with the initiating group and the coordinated monomer at the same Al center.

**Scheme 4:**
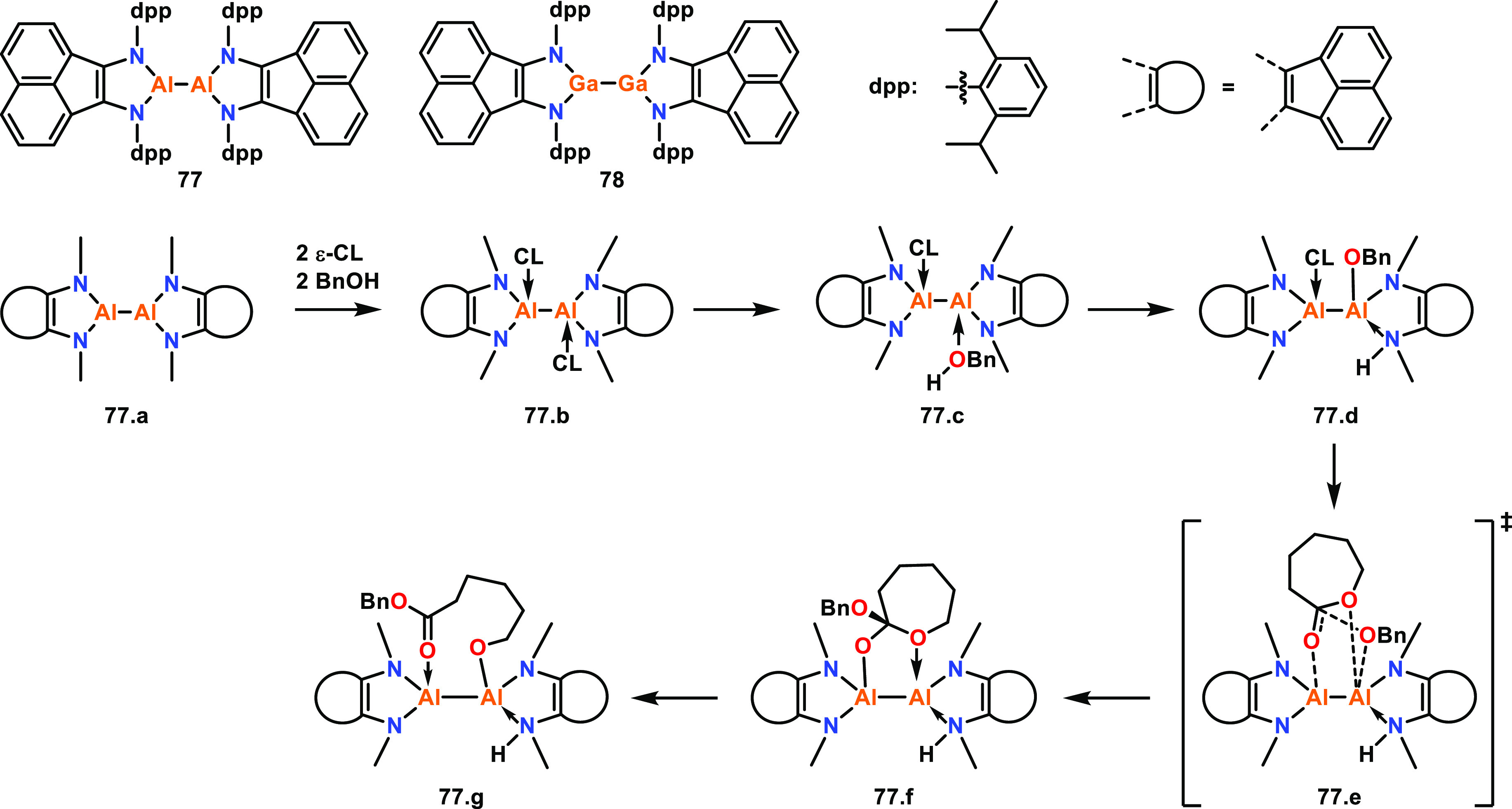
Low Valent Al(II)–Al(II) (**77**) and Ga(II)–Ga(II) (**78**) Metal Complexes and a DFT-Supported Mechanism for the Bimetallic ROP of ε-CL Using the N-Me Analogue of **77** (**77.a**)^63^

Fedushkin, Dagorne and co-workers reported the preparation of low valent Al(II)–Al(II) (**77**) and Ga(II)–Ga(II) (**78**) catalysts for ε-CL ROP and studied the mechanism for the former using density functional theory (DFT) calculations.^63^ While both **77** and **78** could initiate ε-CL ROP at room temperature in the absence of an initiator, this resulted in poor polymerization control, giving higher than expected *M*_n_ and relatively broad dispersities. Addition of 1 equiv of BnOH significantly increased the catalyst activities, and the Al complex **77** was almost 100-fold more active than the less Lewis acidic Ga analogue (**78**, TOF 400 vs 4.2 h^–1^) under the same conditions (RT, toluene, [ε-CL]:[Cat]:[BnOH] 100:1:1). As most Al-based catalysts require heating to reach high ROP activities, the high TOF values observed with **77**/BnOH at RT were remarkable and suggested possible cooperative interactions between the two metal centers. Upon investigation, it was found that when 2 equiv of ε-CL are mixed with **77**, a bis-adduct is obtained where each Al center coordinates a single ε-CL monomer. The adduct was formed quantitatively, and the molecular structure was determined by XRD and NMR spectroscopy. DFT studies were performed to rationalize the high activities observed with the **77**/BnOH system, with the N-dpp (diisopropylphenyl) groups substituted with N-Me for ease of calculation (**77.a**, Scheme 4). The formation of bis-adduct **77.b** was found to be thermodynamically favored (Δ*G* = −12.0 kcal mol^–1^). BnOH displaced one of the monomers, resulting in BnOH coordination at an Al center (**77.c**), and DFT calculations showed that this can trigger an intramolecular proton transfer to a nitrogen of the ligand system. The energy barrier for this transition was low (ΔΔ*G* = 7.3 kcal mol^–1^), and the resulting complex **77.d** was thermodynamically stable (Δ*G* = −33.3 kcal mol^–1^). Nucleophilic attack upon the activated ε-CL subsequently occurred from the neighboring Al-OBn unit through transition state **77.e**. The energy barrier for the formation of **77.f** from **77.d** was calculated to be the highest of all the computed steps (ΔΔ*G* = 27.1 kcal mol^–1^), and is therefore likely to be the rate-determining step. The ring-opening step to form **77.g** was shown to have a low energy barrier (ΔΔ*G* = 0.9 kcal mol^–1^) and forms a thermodynamically more stable product (Δ*G* = −33.3 kcal mol^–1^). These studies demonstrated that cooperative interactions between the Al centers were energetically favorable and rationalized the unusually high ROP activities observed with **77**/BnOH at RT.

**Scheme 5:**
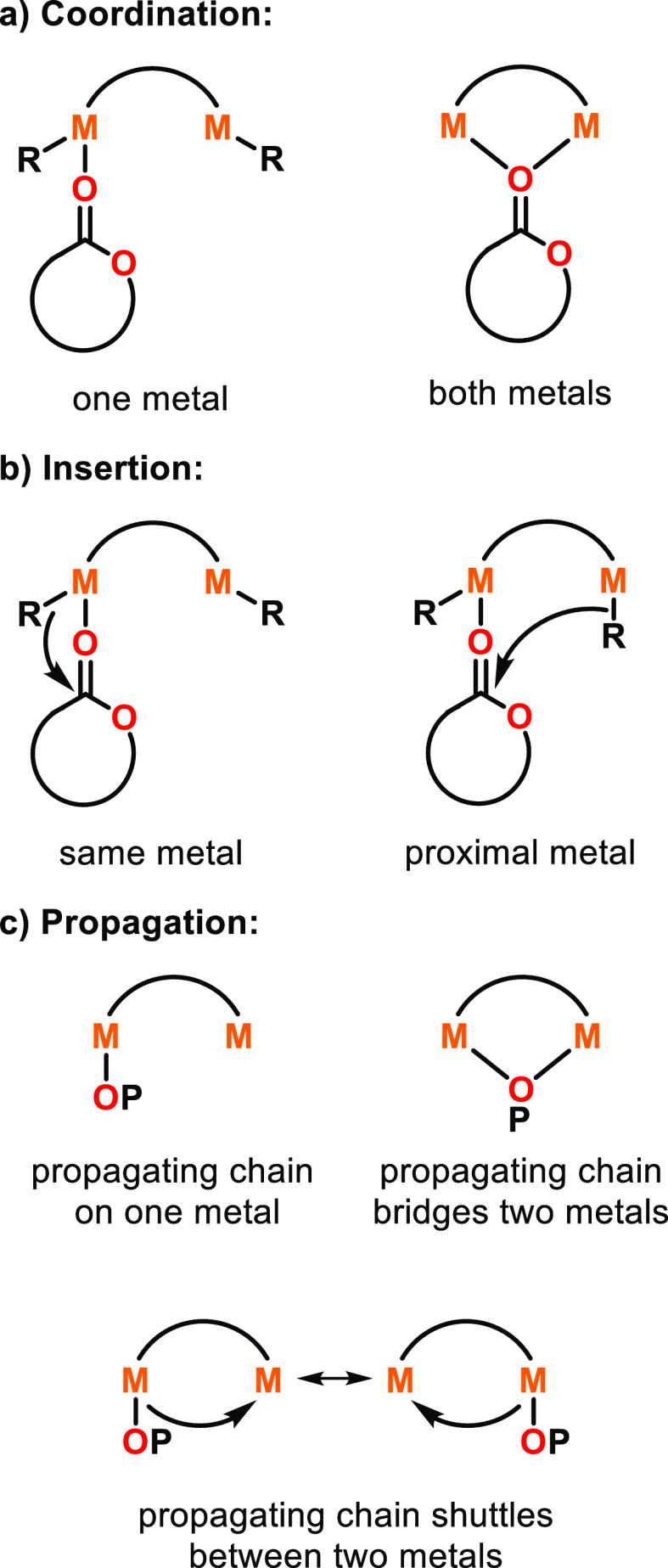
Potential Mechanisms for Cyclic Ester ROP by Bimetallic Catalysts

Coordination–insertion-inspired mechanisms have also been proposed for dizinc catalysts with short Zn–Zn distances of ∼3 Å (refer to Figure 9, section 2.1.2 for details), where the cyclic ester monomer coordinates to both zinc centers simultaneously (Scheme 5).^56^ Taken together, the studies described in this section indicate that while multimetallic catalysts may often follow a CIM, these can differ for different catalysts, varying in whether:

(i) the monomer is activated through coordination to one or two metals (Scheme 5a);
(ii) insertion occurs from an alkoxide on the same metal as the coordinated monomer, or from an adjacent metal-alkoxide (Scheme 5b);
(iii) the propagating chain consistently remains on the same metal, shuttles between the two metal centers, or bridges between the two metal centers (possibly until another monomer is coordinated) (Scheme 5c).

It is important to highlight that this list is not exhaustive, and further mechanistic studies on multimetallic complexes would be useful for establishing trends and informing future catalyst design.

### 2.2 Homotrimetallic and Homotetrametallic Complexes

While multimetallic catalyst development has largely focused on bimetallic catalysts (section 2.1), there are also some reports of tri- and tetrametallic systems, most of which are based on Mg,^64^ Al,^36,53,65,66^ Ga,^67^ Ti,^68,69^ Zn,^70−74^ Zr,^69^ and rare earth metals.^75,76^ Rather than providing a comprehensive review, this section focuses on examples that display multimetallic cooperativity or have delivered insight to the polymerization mechanism and are based on tri- and tetranucleating ligand scaffolds, whereas aggregates are discussed in section 2.4.

**Figure 14:**
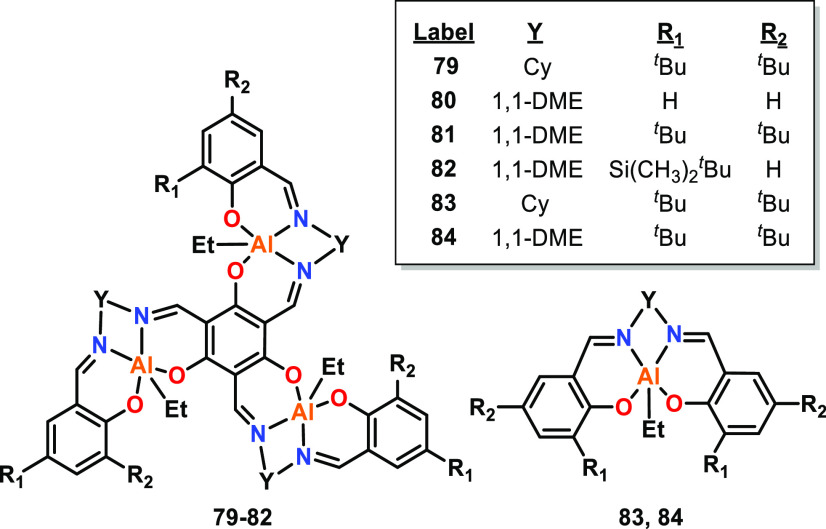
Trimetallic Al complexes **79**–**82** and monometallic Al analogues **83** and **84**, where Cy is cyclohexyl and 1,1-DME is dimethylethylene. Trimetallic **79**–**82** gave significantly higher activities than those of monometallic counterparts **83** and **84** in *rac*-LA ROP.^36^

Chen and co-workers reported four trimetallic Al catalysts (Figure 14, **79**–**82**) based on a tris-salen ligand with various diimine linkers and 2-/4-phenol substituents.^36^ XRD analysis of **81** showed the complex to be bowl shaped, featuring three Al centers in a triangular arrangement with Al–Al distances of 6.38 and 6.40 Å (depending on which complex in the crystal is analyzed), and Al–Al–Al angles of 60.0° in all cases. Trimetallic **79** and **81** gave propagation rate constants (*k*_p_) 133- and 1125-times greater than that of their respective monometallic analogues, **83** and **84** (70 °C, toluene, albeit under different catalyst loadings).^77^ Catalyst **80** gave the fastest polymerization rate of the six complexes (*k*_p_ = 15.4 L mol^–1^ min^–1^, [*rac*-LA]:[Cat]:^*i*^PrOH 600:1:3), and the decreasing rate from **80** > **81** > **82** was ascribed to the increasing steric bulk of the ligand substituents. Three equivalents of ^*i*^PrOH was used to activate all three Al centers for polymerization, and thus three polymer chains were grown per catalyst. The monometallic and trimetallic systems all gave isoselective PLA, ranging from *P*_m_ = 0.64 for **80** to *P*_m_ = 0.98 for **82** (25 °C). The spatial arrangement of the Al centers, asymmetry of the three salen subunits and electronic communication between the three Al centers were suggested to be key factors in the trimetallic systems, delivering enhanced activity and stereocontrol.

**Figure 15:**
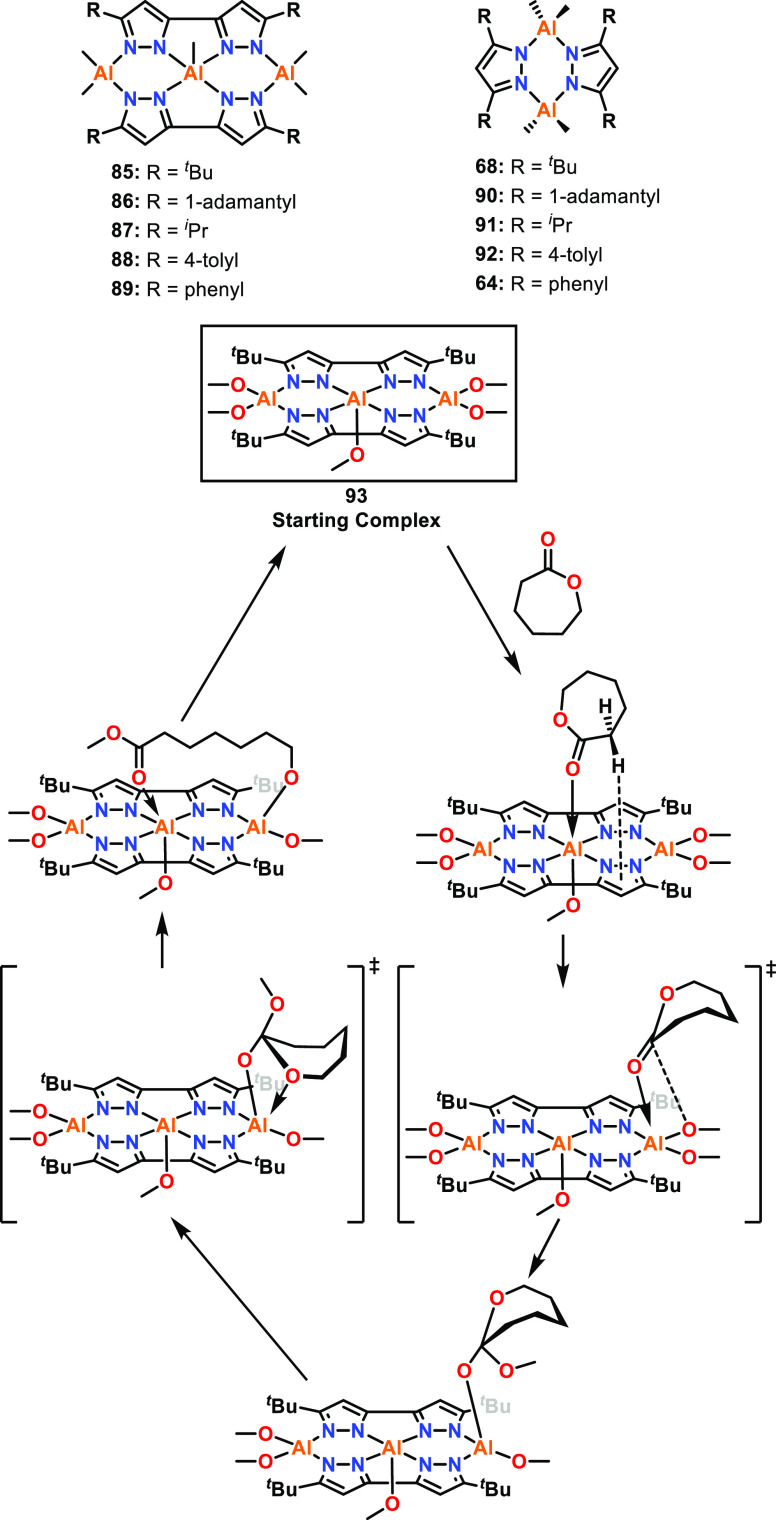
Bimetallic and trimetallic Al catalysts and the mechanism for ROP of ε-CL determined by DFT for complex **93**.^65^

Trimetallic aluminum catalysts featuring three Al centers in a near linear arrangement were reported for ε-CL ROP (**85**–**89**, Figure 15) and showed a trend of increased catalytic activity with the increased steric bulk of aliphatic substituents, from ^*i*^Pr (**87**) < ^*t*^Bu (**85**) < adamantyl (**86**) (25 °C, toluene, [ε-CL]:[Cat]:[BnOH] 100:0.5:2.5).^65^ The trimetallic ^*t*^Bu- and phenyl-substituted complexes both outperformed their bimetallic counterparts (**85** and **89** vs **68** and **64**), with the *k*_obs_ value of **85** being 15 times greater than that of **68**, and **89** being twice that of **64**. XRD studies were performed on trimetallic **85**, which showed Al–Al distances of 3.66 and 3.79 Å and an Al–Al–Al angle of 177.0°. To gain insight into the mechanism, DFT studies were performed on the methoxy analogue of complex **85** (complex **93**), to imitate the active catalyst formed upon alcohol addition, and Mulliken charges were calculated for **85** and **68**. The different Mulliken charges of +0.827 and +0.846 for the terminal Al and +0.929 for the central Al of **85** highlight the different Lewis acidities of the Al centers, especially for the central Al. The Al centers of bimetallic **68** had calculated Mulliken charges of +0.8517, similar to the terminal Al of **85**. Therefore, in the trimetallic system, ε-CL coordination is facilitated by the enhanced Lewis acidity of the central Al, possibly in addition to steric availability due to the square pyramidal geometry leaving a vacant coordination site. Prior to nucleophilic attack, ε-CL transfers to a terminal Al center, with subsequent nucleophilic attack occurring from a terminal Al-OMe unit. After the ε-CL monomer is ring-opened, it bridges between the terminal Al (Al-alkoxide) and the central Al (ester coordination). The Lewis donor coordination at the central Al center can subsequently be displaced, enabling coordination of another ε-CL molecule and continuation of the catalytic cycle. This proposed catalytic cycle gives strong evidence for the cooperativity of the metal centers though electronic modulation and metal–metal proximity, leading to synergistic effects.

**Figure 16:**
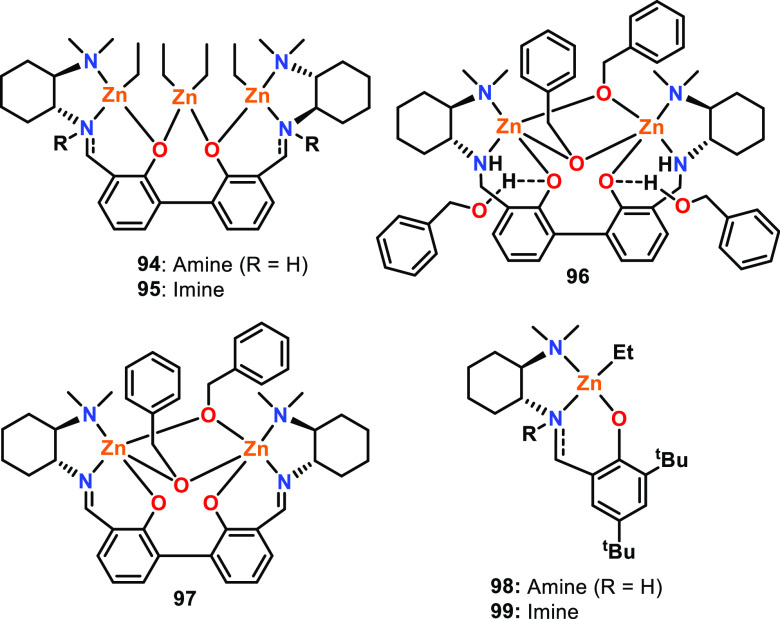
Mono-, bi-, and trimetallic Zn aminophenolate and iminophenolate complexes tested for *rac*-LA ROP, where aminophenolate complexes **94**, **96**, and **98** outperformed their respective iminophenolate counterparts **95**, **97**, and **99** and the bi- and trimetallic complexes outperformed the monometallic analogues.^73^

Mehrkhodavandi and co-workers reported a series of trimetallic Zn complexes based on aminophenolate and iminophenolate ligands for the ROP of *rac*-LA, which were compared to the bimetallic and monometallic analogues (**94**–**99**, Figure 16; see also section 2.1.3, Figure 11 for additional discussions of the Zn-benzoxide analogues of **98** and **99**).^73^ Single crystal XRD studies were performed on trimetallic **94** and bimetallic **96** and **97**. For **94**, the Zn–Zn distances were 3.60 and 3.62 Å with a Zn–Zn–Zn angle of 142.2°, whereas bimetallic **96** and **97** showed shorter Zn–Zn distances of 2.91 and 2.87 Å, respectively. Polymerization studies (25 °C, CH_2_Cl_2_, [*rac*-LA]:[Zn] 600:1) showed that aminophenolate complex **94** was faster than iminophenolate complex **95**, giving 98% conversion (*Đ* = 1.3) in just 4 h compared to 78% (*Đ* = 1.2) in 24 h, respectively. A similar observation was made for bimetallic **96** and **97** (RT, CH_2_Cl_2_, [*rac*-LA]:[Zn] 600:1), with aminophenolate complex **96** giving 97% conversion (*Đ* = 1.2) at 5 h compared to 89% (*Đ* = 1.1) at 24 h for complex **97**. Monometallic Zn-Et catalysts showed the same trend but were much less active, with the amino complex **98** converting 72% of *rac*-LA after 44 h compared to 46% conversion with the imino analogue **99** (25 °C, CH_2_Cl_2_, [*rac*-LA]:[Zn] 200:1). While the bimetallic and trimetallic systems outperformed the monometallic analogues, implying multimetallic cooperativity, the authors highlighted that this difference may be due to other factors such as the steric encumbrance of the ligand.

Far fewer tri- and tetrametallic catalysts have been reported than bimetallic complexes for LA and ε-CL ROP, yet tri- and tetrametallic systems (and beyond) offer an opportunity to vary the three-dimensional arrangement of the metal centers (e.g., from linear to triangular geometries), which may impact catalyst performance. However, it is important to note that while some tri- and tetrametallic systems have shown enhanced activities, others have not, and some have not been benchmarked against the monometallic analogues to investigate the potential for multimetallic cooperativity. While this research is at a relatively early stage compared to bimetallic catalyst development, similar factors appear to be important, including the M–M proximity, electronic communication between the metal centers, and the steric accessibility of the metal centers.

### 2.3 Heterometallic Complexes

In addition to homometallic complexes, increasing numbers of heterometallic complexes have been reported as highly active catalysts for cyclic ester ROP.^16^ Heterometallic complexes are attractive because each metal can be tailored toward a specific role, by providing different metal sites for the key mechanistic steps of monomer activation and nucleophilic attack (refer to section 2.1.4). Therefore, heterometallic complexes have the potential to further extend multimetallic cooperativity beyond what is possible with homometallic complexes.^78,79^ A vast number of different heterometal combinations are available, and a variety of cooperative heterometallic systems have been reported from across the periodic table.^6^ While many of these heterometallic complexes have shown superior activity for ROP compared to the homometallic analogues, other heterocombinations remain unexplored. It is also important to note that some heterometallic catalysts display poorer performance than the homometallic analogues.^33,80^ As recent articles have comprehensively reviewed heterometallic catalysts for cyclic ester ROP,^16,31^ here we focus on catalysts that display heterometallic cooperativity and deliver insight into the structure/activity relationships compared to their homometallic counterparts.

To the best of our knowledge, no detailed mechanistic studies have been reported for heterometallic catalyzed cyclic ester ROP. However, some key experimental trends are starting to emerge. For example, enhanced activities are typically observed when a relatively electronegative metal (e.g., Zn) is combined with a medium/large and Lewis acidic metal such as an alkali metal (K/Na) or lanthanide.^16,81−83^ Larger group 1 or lanthanide metals have typically displayed enhanced catalyst activities, attributed to the presence of additional monomer coordination sites.

It is challenging to provide direct comparisons between homo- and heterometallic catalysts, as the heterometallic systems contain more than one metal (i.e., are bimetallic or trimetallic) but are typically compared to the monometallic analogues. This often introduces variation in the metal coordination environments, due to differences from the ligand (e.g., the number of ligands and the presence of bridging or terminal coligands), the metal oxidation state, and/or the number of initiating groups per metal center. In some cases, different mechanisms are available depending on the number of initiating units and the ratio of catalyst:co-initiator. Furthermore, not all heterometallic catalysts retain their structures in solution.^81^ Therefore, benchmarking against directly comparable species and analysis of the solution-state structures can provide key insights into whether or not the enhanced performance is truly due to heterometallic cooperativity.

**Figure 17:**
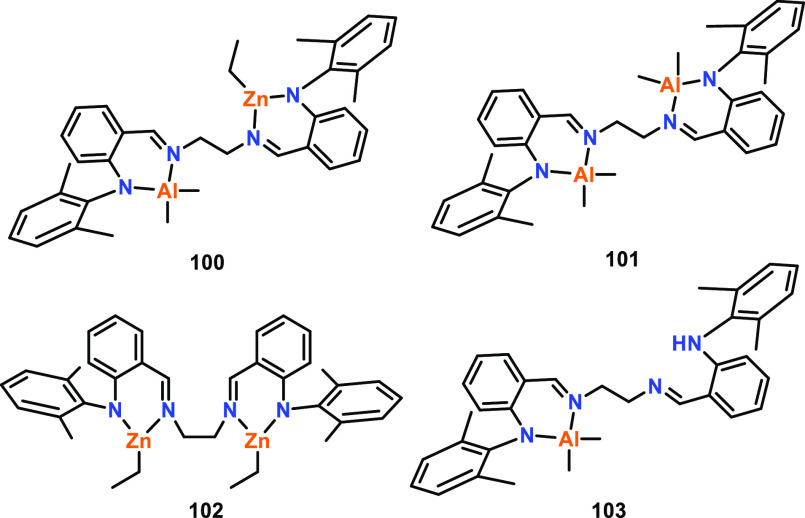
Schiff base ligand-derived Al and Zn complexes. Tested for ε-CL ROP, heterometallic **100** outperformed mono- and bis-Al catalysts **101** and **103** yet gave lower activities than bis-Zn catalyst **102**.^84,85^

Within ROP, very few heterometallic catalysts have been directly compared to their homobimetallic analogues. Mu and co-workers reported a heterobimetallic Al/Zn complex **100** along with the homobimetallic analogues **101** and **102** and monometallic **103** (Figure 17); these complexes were all active catalysts for ε-CL ROP in the presence of BnOH.^84,85^ Heterometallic **100** was more active than the mono-Al and bis-Al counterparts (**103** and **101**), resulting in 95% conversion in 6 min compared to 94% in 5.5 h or 92% in 20 min, respectively (70 °C, toluene, [ε-CL]:[Cat]:[BnOH] 100:1:2). However, **100** was slower than the bis-Zn analogue **102**, which produced 98% PCL in 1 min under the same conditions. The higher catalytic activity of **102** was attributed to the lower bond dissociation energy of the M–O bond (284 kJ mol^–1^ for Zn–O vs 512 kJ mol^–1^ for Al–O). The bimetallic catalysts were determined to proceed via a CIM, where the active catalyst was produced by alcoholysis of the metal–alkyl complex with BnOH. The BnOH stoichiometry influenced the activity of the bimetallic catalysts and the molar mass of the resultant PCL; *M*_n_ decreased with additional BnOH. For heterometallic **100** and the bis-Zn analogue **102**, the highest activity was achieved using a [ε-CL]:[Cat]:[BnOH] loading ratio of 100:1:2, whereas the bis-Al system **101** performed best with a ratio of 100:1:1, although the reason for this difference remains unclear.

**Figure 18:**
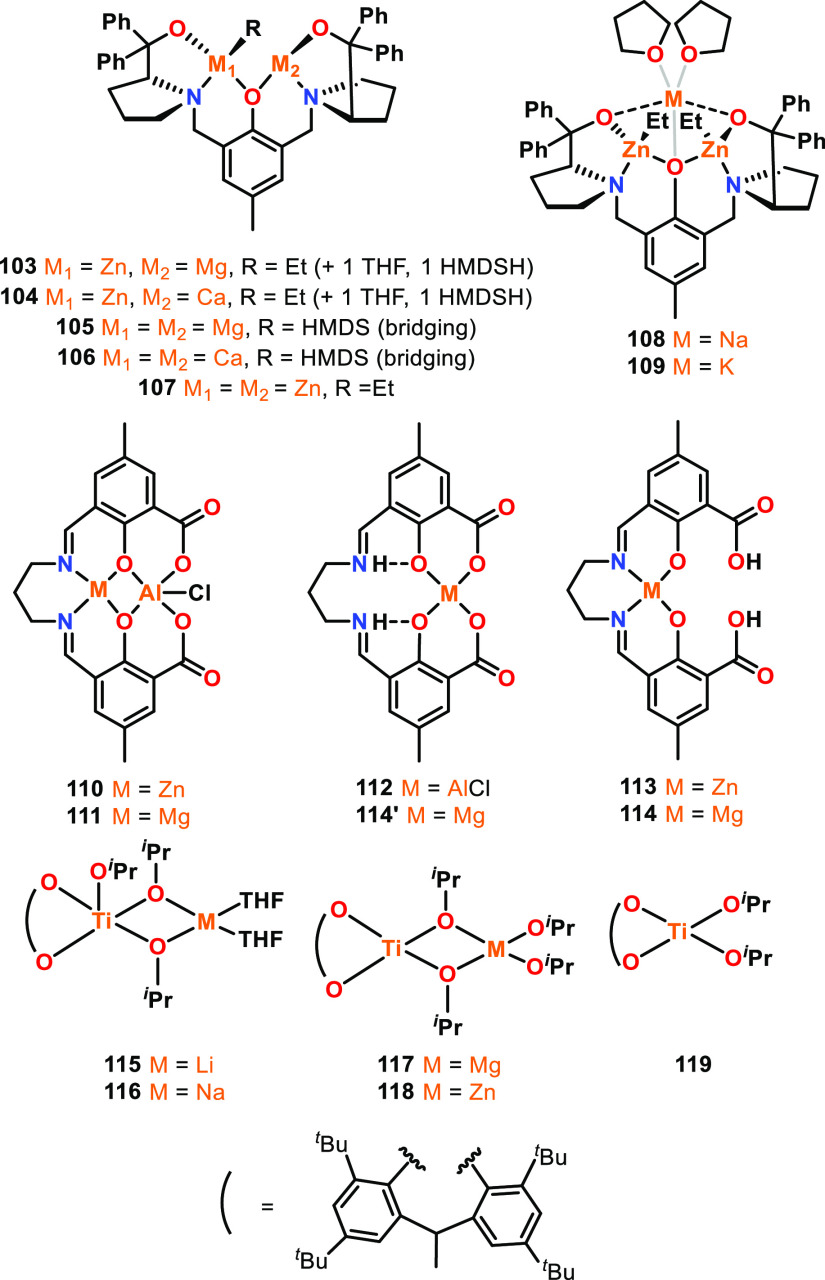
Examples of heterometallic catalysts for cyclic ester ROP, with **114** and **114′** highlighting different metalation sites observed for Mg. For complexes **103**–**107**, heterometallic **104** displayed the highest activity for *rac*-LA ROP; complex **109** outperformed **108** in *rac*-LA ROP; heterometallic **110** and **111** outperformed monometallic **112**–**114′** in *rac*-LA ROP (**113**-**114′** were inactive); heterobimetallic complexes **115**–**118** all outperformed monometallic **119** for l-LA ROP.^33,88,89,93^

In complexes **100**–**102**, the two metals are separated by an ethylenediamine bridge, yet most heterometallic catalysts for cyclic ester ROP feature two metals directly bridged by an alkoxide. This bridging M–O–M′ framework can result in “ate”-type structures, which can simultaneously enhance monomer coordination at one metal (M) and the reactivity of the other M′–R bond toward nucleophilic attack or deprotonation.^86−89^ Highly active heterometallic Mg/Zn **103** and Ca/Zn **104** catalysts have been reported that contain only divalent metals, thus enabling a direct comparison to the homometallic analogues (bis-Mg, bis-Ca, and bis-Zn, **105**–**107**).^88^ The Ca/Zn complex **104** exhibits the highest activity (Figure 18), giving 82% conversion of *rac*-LA in 1.25 min and outperforming the bis-Zn **107** and bis-Ca analogues **106**, which gave respective conversions of 27% and 64% under the same conditions (60 °C, toluene, [*rac*-LA]:[Cat]:[BnOH] 100:1:1). Heterometallic **104** also outperformed the Mg/Zn analogue **103** (82% vs 25% conversion after 1.25 min), although **103** was more active than bis-Mg **105** (84% vs 66% *rac*-LA conversion at 10 min) and gave similar activities to bis-Zn **107** (87% conversion in 10 min). DFT studies showed the coordination of THF and HMDSH to the group 2 metals (rather than Zn), and the larger Ca(II) provides a greater number of coordination sites. Therefore, Mg and Ca were proposed to coordinate the monomer, with Zn acting as the source of the nucleophile. As the periodic table contains a diagonal relationship in ionic radius,^90,91^ with Na and Ca bearing similar ionic radii, trimetallic Na/Zn_2_ and K/Zn_2_ catalysts have also been reported based on the same prophenol ligand (**108**–**109**, Figure 18).^89^ In the presence of 2 equiv of BnOH, **108** and **109** converted 47 or 60 equiv of *rac*-LA in just 20 s at RT, with respective *k*_obs_ values of 3.2 × 10^–3^ s^–1^ and 1.7 × 10^–2^ s^–1^ (THF, [*rac*-LA]:[Cat] 100:1). The larger and more electropositive metal (Na or K) was proposed to act as the monomer coordination site, with Zn providing the source of the metal-alkoxide nucleophile.^89,92^ While these complexes are trimetallic rather than bimetallic, the same trends based on ionic radius and electronegativity difference between the metals were observed, with larger and more electropositive heterometals leading to enhanced catalyst activities (K/Zn_2_ > Na/Zn_2_ > CaZn > MgZn). Incorporating Na/K also labilized the Zn–Et bonds (vs the bis-Zn complex), as evidenced by an upfield shift of the Zn–C*H*_2_ resonance in ^1^H NMR spectroscopy, which was proposed to accelerate the nucleophilic attack and LA ring-opening.

Introducing a heterometal has also increased the nucleophilicity of Al–Cl initiating units in Zn/Al and Mg/Al salen complexes (complexes **110** and **111**, Figure 18),^33^ which reacted with propylene oxide (PO) in situ to form an active Al-alkoxide.^94^ Both **110** and **111** displayed good activities in *rac*-LA ROP, outperforming mono-Al **112**, with respective *k*_obs_ values of 1.8 × 10^–3^ s^–1^, 8.8 × 10^–3^ s^–1^, and 0.8 × 10^–3^ s^–1^ (120 °C, toluene, [*rac*-LA]:[Cat]:[PO] 100:1:50). In contrast, the mono-Zn and mono-Mg analogues (**113** and **114/114′** mixture) were completely inactive under the same conditions. DFT studies showed that the chloride initiating unit forms a dative interaction to the heterometal, bridging between the two heterometals and thus elongating and weakening the Al–Cl bond, which correlates to a shorter initiation period and faster propagation rate. This highlights that multimetallic cooperativity can influence the coligand to enhance both the initiation and propagation stages of ROP. Intriguingly, this study revealed that some of the well-established catalyst trends for monometallic salen ROP catalysts are reversed with heterometallic salen catalysts. For instance, with monometallic salen catalysts, flexible ligand scaffolds generally give improved activity, attributed to the ease with which key transition states can be accessed.^57^ In contrast, with heterometallic **110** and **111**, the more rigid catalysts displayed the highest catalyst activities (Mg/Al > Zn/Al > Al).

Efficient heterometallic ROP catalysts featuring transition metals have also been reported, including a series of heterobimetallic M/Ti(IV) initiators **115**–**118** (M = Li, Na, Mg or Zn), which all outperformed the monometallic Ti initiator **119** for l-LA ROP.^93^ Complexes **118** and **117**, featuring divalent Zn and Mg, were especially active and converted 91% and 89% of l-LA within 30 min and 3.5 h, respectively (both with *Đ* = 1.3), whereas alkali metal complexes **115** and **116** required 94 h for 74–80% conversion (30 °C, toluene, [l-LA]:[Cat] 100:1 for **118** and **117**, and [l-LA]:[Cat] 150:1 for **115** and **116**). The enhanced activity of **118** compared to **117** was attributed to differences in the electronic configurations and charge densities of Zn and Mg, with the charge density of Mg > Zn resulting in a stronger Mg–OR bond and a decreased polymerization rate.

**Figure 19:**
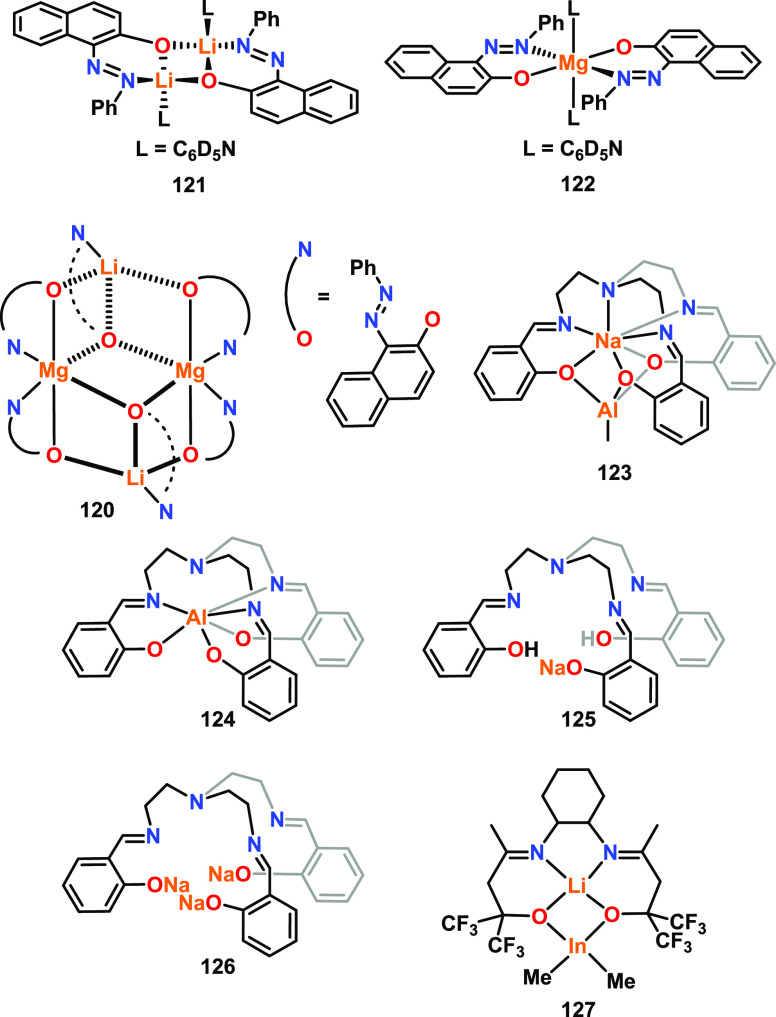
Further examples of heterometallic complexes for cyclic ester ROP centered on group 1 and 2 metal combinations. Bimetallic **121** outperformed monometallic **122** and heterometallic Mg/Li **120** for *rac*-LA and l-LA ROP; mono- and trimetallic sodium complexes **125** and **126** outperformed heterometallic **123** and homometallic aluminum **124** in *rac*-LA ROP (**124** was inactive); heterometallic **127** displayed good activity in *rac*-LA ROP.^80,95,96^

While most of the reported heterometallic catalysts follow a CIM, some follow an AMM. For example, heterometallic Li/Mg complex **120** was tested for *rac*-LA and l-LA ROP and benchmarked against the homometallic analogues **121** and **122** using BnOH as a co-initiator (Figure 19).^95^ Many ROP catalysts include an imine group (e.g., salen scaffolds); complexes **120**–**122** instead feature an azo functionality, where the weaker sigma electron donation properties (vs imine) were designed to boost the Lewis acidities of the metals and enhance monomer coordination. Mechanistic studies were performed by ^1^H NMR monitoring a 1:1:1 mixture of the catalyst, BnOH, and l-LA. Complexes **120** and **121** remained intact while l-LA was inserted into BnOH, indicating that BnOH acted as an exogeneous initiator and the reaction followed an AMM. In contrast, the Mg complex **122** indicated a possible ligand-assisted CIM, as the protonated 1-phenylazo-2-naphthoxo ligand was observed. For both l- and *rac*-LA ROP, bis-Li **121** was more active than mono-Mg **122**. For example, **121** gave 90% conversion of *rac*-LA in 0.5 h at 25 °C, whereas **122** required longer reaction times and elevated temperature to achieve 83% conversion (7 h, 70 °C, toluene [LA]:[metal]:[BnOH] = 100:1:1). While different reaction temperatures were used, the activity of heterometallic **120** appears to be intermediate between homometallic **121** and **122**, reaching 94% conversion in 8 h at 25 °C ([LA]:[metal]:[BnOH] = 100:1:1).

A heterometallic Na/Al complex based on the TrenSal ligand was also reported to follow an AMM for *rac*-LA ROP (**123**, Figure 19).^96^ While the monometallic Al complex **124** was completely inactive under identical conditions, the mono- and trimetallic Na analogues **125** and **126** displayed higher activities than that of heterometallic **123**. Trimetallic **126** was the most active (*k*_obs_ = 1.21 min^–1^) but with poor polymerization control (RT, toluene, [*rac*-LA]:[Cat]:[BnOH] 100:1:1), a combination that is often observed with alkali metal catalysts in LA ROP.^27,30^ Heterometallic **123** offers a balance between activity and control, displaying good activity (vs the inactive Al complex **124**) and improved control compared to Na complexes **125**–**126** (*Đ* = 1.5 for **123** vs 1.8 for **125** and 2.0 for **126**. Small molecule reactivity studies monitored by ^1^H NMR analysis suggested that polymerization with **123**/BnOH or **125**/BnOH follows an AMM, whereas a combination of AMM and CIM occurs simultaneously for **126**. Similarly, an AMM was proposed for the heterometallic Li/In complex **127**.^80^ Complex **127** displayed good activities for *rac*-LA ROP with and without 1 equiv of ^*i*^PrOH, converting 98 and 96 equiv of *rac*-LA in 30 and 60 min, respectively (80 °C, toluene, [*rac*-LA]:[Cat] 100:1), albeit with low polymerization control in both cases (*Đ* = 2.6 and 2.2, respectively).

**Figure 20:**
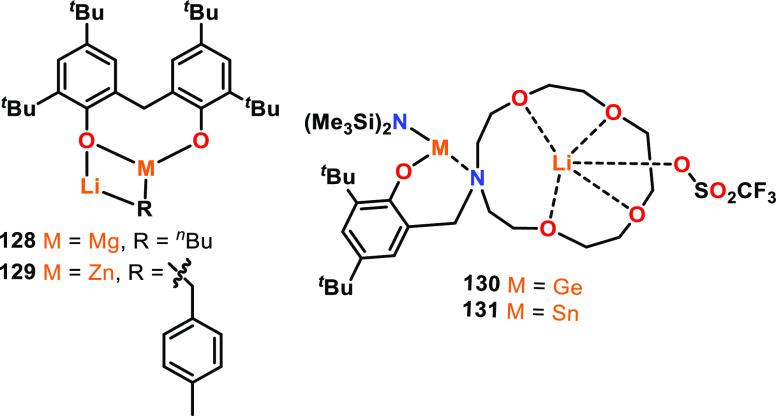
Li-containing heterometallic complexes for LA ROP.^97,98^

Exogeneous alcohols such as BnOH can activate heterometallic catalysts toward ROP, either through alcoholysis to convert a metal-alkyl group to an active metal-alkoxide (CIM), or by itself acting as the nucleophile (AMM). However, recent studies have shown that BnOH can also rearrange heterometallic catalyst structures, and that solution-state studies are thus of key importance to understand how structural changes can affect catalyst activity in ROP.^81^ Solvent choice can also significantly influence heterometallic solution-state structures. For instance, the in situ-generated Li/Mg and Li/Zn complexes **128** and **129** reported by Thomas and co-workers (Figure 20, left) were both active for *rac*-LA ROP at RT using 1 equiv of neopentyl alcohol, yet displayed different activities and control in coordinating and noncoordinating solvents, specifically toluene and THF.^97^ Using a mixed toluene/THF solvent system for **128** delivered both high activity and stereocontrol, reaching full conversion after 15 min with *P*_r_ = 0.84. In contrast, using solely THF reduced the stereocontrol (*P*_r_ = 0.54) whereas using toluene alone reduced the activity (43% conversion at 45 min). The use of Lewis donor solvents is widely known to alter the aggregation states of organometallic complexes (refer to section 2.4 for details). Ligand scaffolds that incorporate Lewis donors, such as crown ethers, have therefore been used to support heterometallic complexes.^98^ For example, Sarazin and co-workers investigated the activities of Li/Ge **130** and Li/Sn **131** complexes (Figure 20, right) in LA ROP and compared these to the monometallic Ge and Sn analogues. Although the Li/Sn complex was slower than the mono-Sn analogue, the Li/Ge complex was almost twice as active as the mono-Ge complex, giving 57% l-LA conversion (vs 35%) with good polymerization control (*Đ* = 1.1, 100 °C, toluene, [l-LA]:[Cat]:[^*i*^PrOH] 500:1:10). The low activity of the heterometallic Li/Sn complex was attributed to greater air- and water-sensitivity and possible decomposition during ROP.

**Figure 21:**
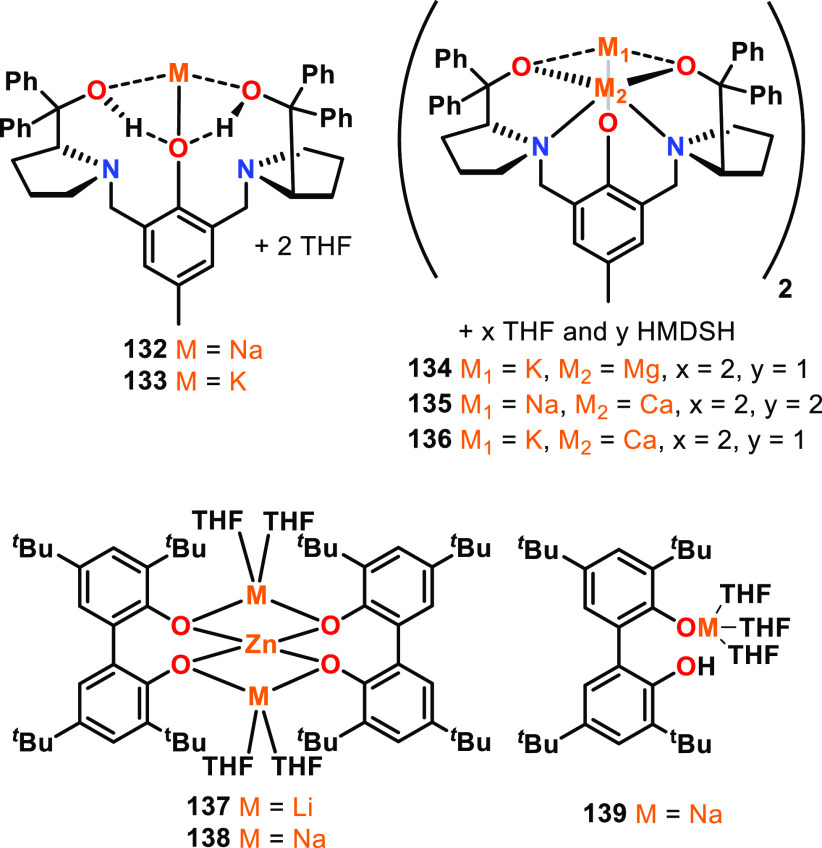
Examples of homo- and heterometallic complexes for *rac*-LA ROP (**132**–**136**) or l-LA ROP (**137**–**139**).^81,99^

While various methodologies are available for preparing heterometallic complexes, including sequential deprotonation, coordination, and/or transmetalation routes, the isolation of heterometallic complexes can be synthetically challenging. Recently, simple strategies to target heterometallic cooperativity have been reported. For example, the simple in situ combination of a monometallic alkali metal complex (Figure 21, **132** or **133**) with a metal benzoxide salt (Mg(OBn)_2_, Ca(OBn)_2_ or Zn(OBn)_2_) gave high conversions of *rac*-LA (50–89%) in just 5 s, albeit with moderate control over the dispersities (*Đ* = 1.2–2.0).^81^ Notably, almost all of these combinations delivered enhanced activity compared to the monometallic alkali metal complex or the metal benzoxide salt when tested separately. Solution-state analysis of these catalyst systems by DOSY NMR revealed a complex mixture of species were present. Intriguingly, detailed NMR studies indicated that a similar mixture of species was generated when the isolated heterometallic complexes (Figure 21, **134**–**136**) were combined with BnOH. Furthermore, similarly high activities were observed for LA ROP whether the catalyst system was generated from complex **132** and **133** with a metal benzoxide salt, or from the isolated heterometallic catalyst **134**–**136** and BnOH. These observations indicate that heterometallic activity enhancements can be harnessed without synthetically isolating heterometallic complexes, and that the solution-state structures of heterometallic catalysts can be complex.

Most well-defined metal initiators are air- and moisture-sensitive. As this requires specialist handling techniques and anhydrous reaction conditions, developing robust, air- and moisture-tolerant systems is an attractive target. Accordingly, in 2011, Wu and co-workers published two heterometallic Li/Zn and Na/Zn catalysts that were designed to be air-stable, with Li–Zn distances of 2.75 and 2.79 Å and Na–Zn distances of ∼3.03 Å (**137** and **138**, Figure 21).^99^ Complexes **137** and **138** were both active for l-LA ROP, giving respective conversions of 91% and 90% after 48 h with reasonable dispersities of *Đ* = 1.4–1.5 (90 °C, toluene, [l-LA]:[Cat] 150:1). Good activities were retained upon increasing the [l-LA]:[Cat] ratio to 250:1. It is worth noting that these polymerizations were performed without an exogenous alcohol, and that Zn-phenoxide units would be expected to be relatively poor initiators compared to Zn-alkoxide catalysts featuring more labile Zn–O bonds. However, **138** was active for l-LA ROP even when the reactions were performed in air using an unpurified monomer source, displaying high conversions of 94% after 48 h with controllable molar mass (*Đ* = 1.4, 90 °C, toluene, [l-LA]:[Cat] 175:1). Additionally, **138** still polymerized l-LA with 85% conversion after 48 h in the presence of 50 equiv of water (*Đ* = 1.3). This was attributed to complex **138** hydrolyzing to form monometallic **139**, which was also an active catalyst for l-LA ROP (>90% conversion, 24 h, 90 °C, toluene, [l-LA]:[Cat] 250:1).

### 2.4 Aggregate Catalysts for ROP

While many multimetallic systems use ligand scaffolds to encapsulate multiple metals in close proximity, multimetallic catalysts can also be formed through the aggregation of individual molecules. Within ROP, aggregated catalysts are typically based on homometallic Li,^62,100−106^ Mg,^101,107,108^ Al,^109^ In,^110^ or Ti compounds,^111,112^ or heterometallic mixtures thereof.^95,113^ Aggregated compounds can have complex solution-state chemistry, where the aggregate structures observed in the solid state can dissociate or undergo dynamic equilibria in solution, in the presence of Lewis bases (e.g., cyclic ester monomers) and during reactions (e.g., ROP). However, some multimetallic aggregates are highly active ROP catalysts that can deliver desirable and sometimes unexpected polymer properties, such as stereocontrolled PLA using achiral organometallic reagents.^101,105,114^

For example, lithium *tert-*butoxide (LiO^*t*^Bu) can exist as a pentameric aggregate in THF solution and was reported to give PLA with a heterotactic bias, implying that there is a preference for the alternate insertion of d- and l-LA into the polymer chain from *rac*-LA.^105,114,115^ As the O^*t*^Bu ligand is achiral, this indicates that the reaction follows a chain end control mechanism,^116^ where the preference of the next monomer to be inserted into the polymer chain is dictated by the stereochemistry of the previously inserted monomer. Indeed, LiO^*t*^Bu initially generated heterotactic PLA from −20 to 20 °C (THF, [*rac*-LA]:[Cat] 250:1), delivering *P*_r_ values of 0.94 (5.0 min, −20 °C), 0.92 (3.0 min, 0 °C) and 0.90 (0.5 min, 20 °C). However, a loss in tacticity due to transesterification was observed by ^13^C NMR in the later stages of the reaction.

**Figure 22:**
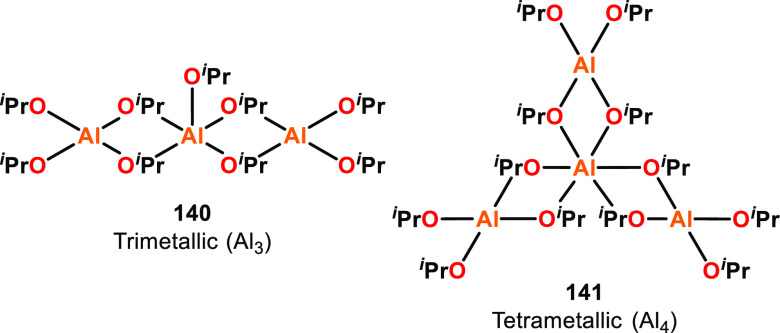
Structures of trimeric and tetrameric aluminum isopropoxide, where trimetallic **140** outperformed tetrametallic **141** in ε-CL and l-LA ROP.^123−125^

Both butyllithium (BuLi) and butylmagnesium (Bu_2_Mg) aggregates can also deliver partial heterotacticity control in *rac*-LA ROP.^101^ BuLi can adopt different aggregation states depending on the solvent and can exist as a hexamer in hexane and a tetramer or dimer in THF.^117−120^ The solid-state structure of Bu_2_Mg has not yet been reported, although crystalline *tert*-butylmagnesium exists as a dimer.^121,122^ While BuLi delivered slightly higher heterotacticity control than that of Bu_2_Mg, transesterification was observed using BuLi at [LA]:[BuLi] ratios of 200:1 or 400:1, (20 °C, hexane/THF), which reduced the tacticity control. Interestingly, no transesterification was observed with Bu_2_Mg under the conditions tested. It is worth highlighting that heterometallic Li/Mg aggregates have also delivered PLA with a heterotactic bias, including Li/Mg **120** (Figure 22, refer to section 2.3 for details).^95^ Notably heterometallic **120** gave greater stereocontrol than that of the homometallic analogues (Li/Mg **120**, *P*_r_ = 0.75; Li **121**, *P*_r_ = 0.67; Mg **122**, *P*_r_ = 0.46). Detailed DOSY investigations revealed that the aggregation state of heterometallic **120** persisted in C_6_D_6_ solution.

To probe the importance of different aggregation states, the Penczek group investigated two aggregates of Al(O^*i*^Pr)_3_, trimeric Al_3_ and tetrameric Al_4_ (**140** and **141**, Figure 22), for the ROP of ε-CL and l-LA.^123,124^ The structure of Al_3_ was computationally calculated and showed that the terminal Al atoms are tetracoordinate while the central Al is pentacoordinate.^123^ The structure of Al_4_ was determined by XRD studies and features a central hexacoordinate Al atom with three peripheral tetracoordinate Al atoms.^125^ For both the polymerization of ε-CL and l-LA, Al_3_ was much faster than Al_4_. For ε-CL; the initiation rate of Al_3_/Al_4_ was 10 000 at 20 °C, and for l-LA, Al_3_/Al_4_ was 4100 at 20 °C, 800 at 50 °C, and 290 at 80 °C in THF solvent.^124^ The reactivity differences between Al_3_ and Al_4_ were ascribed to the different coordination numbers of the central Al atoms in the trimetallic and tetrametallic aggregates. For both polymerization systems, three polymer chains were grown per Al center, indicating that although the aggregation state affected the rate of initiation, disaggregation occurred during propagation. It is worth noting that equilibrium processes were proposed to occur throughout the polymerization, including interconversion between the trimeric and tetrameric species, disaggregation into the active monomeric species during propagation, and reversible aggregation of the propagating monomeric species into unreactive dimers. Overall, these studies highlight the complex equilibria that can occur with solution-state aggregates.

**Figure 23:**
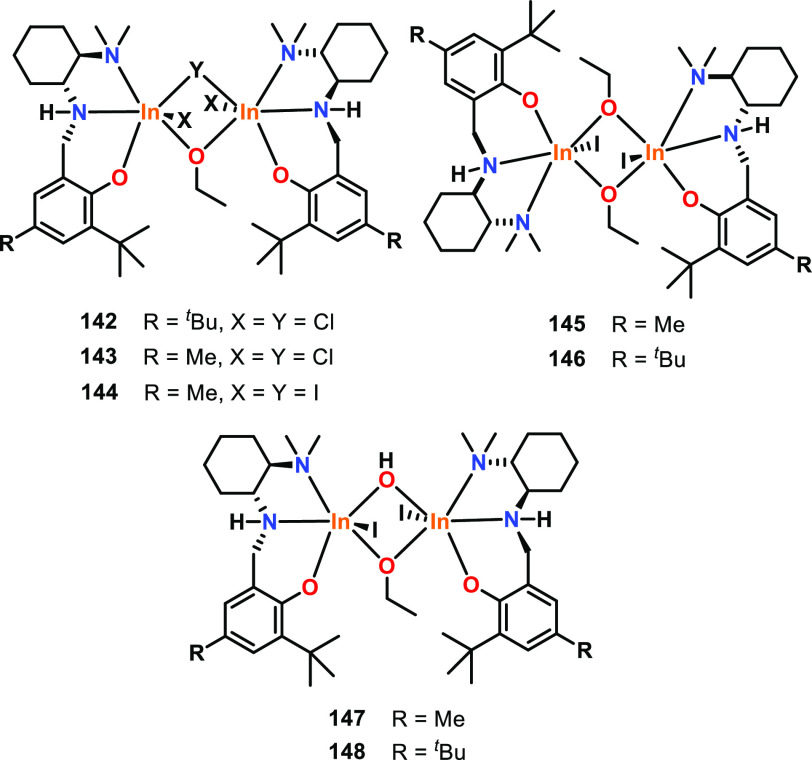
A series of racemic and enantiopure bimetallic indium complexes with sterically demanding diaminoaryloxy ligands tested for LA ROP.^127^

Mehrkhodavandi and co-workers reported detailed investigations into aggregated In complexes.^126^ Bimetallic **142**, featuring chiral diaminoaryloxy ligands, produced high molar mass PLA with narrow dispersities (*M*_n_ > 350 kg mol^–1^ and *Đ* < 1.1, RT, CH_2_Cl_2_, [*rac*-LA]:[initiator] 2100:1). Based on XRD and NMR spectroscopic studies, including variable temperature, 2D nuclear Overhauser effect, and pulsed gradient spin–echo spectroscopy, **142** was shown to exist as a bimetallic aggregate in both the solid- and solution-state (Figure 23).^127^

**Scheme 6:**
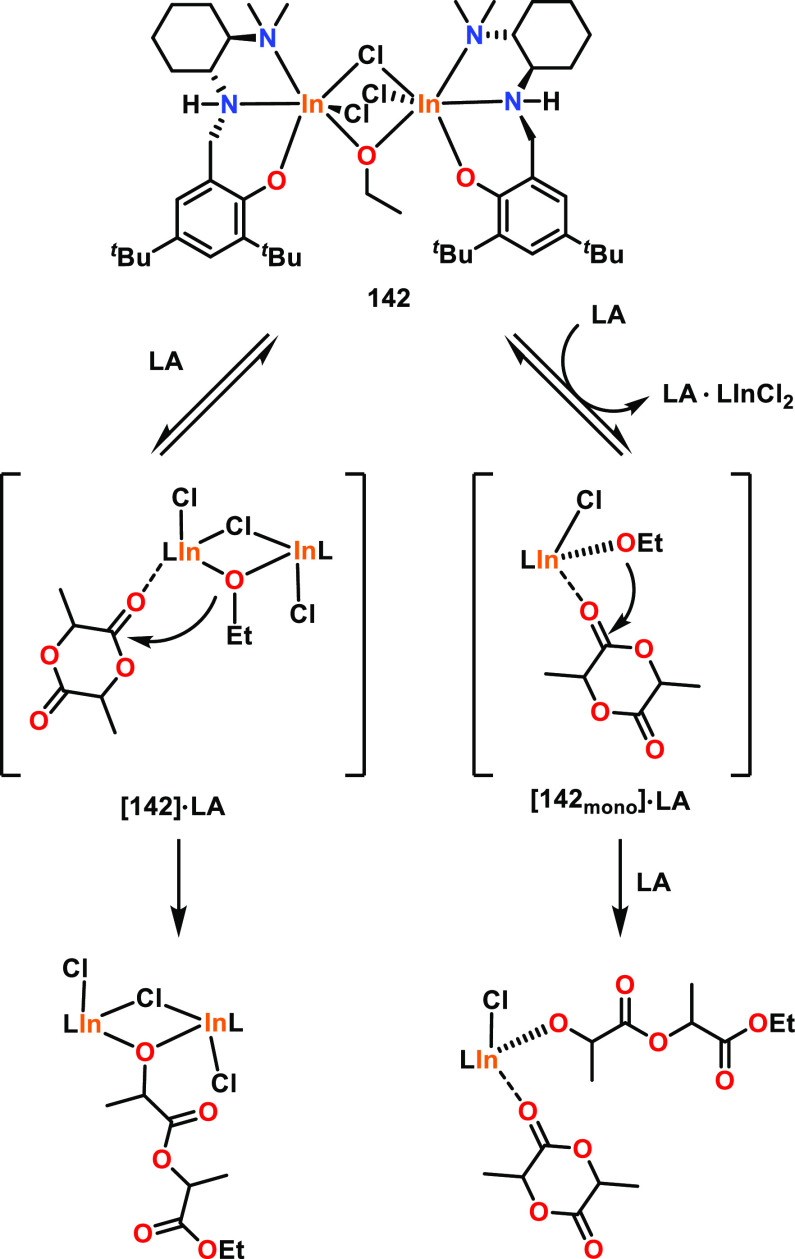
Two Proposed Mechanical Pathways for the ROP of LA Using Complex **142**(127,128)

Two different mechanisms were proposed for the ROP of LA using **142** (Scheme 6).^127^ One involved dissociation of **142** into two different species (Scheme 6, right), an active monometallic species, **[142**_**mono**_**].LA**, and an inactive indium chloride complex. In the other, bimetallic **142** remained intact and both metal centers stabilized the propagating polymer chain (Scheme 6, left). Computational calculations showed that dissociation of dimer **142** is a strongly endothermic process with an energy requirement of 32.1 kcal mol^–1^,^128^ and so **142** has a low dissociation tendency even in the presence of a strong base, such as neat pyridine at 100 °C. These observations supported the bimetallic mechanism (Scheme 6, left), which was further evidenced by studies into the polymerization rates, molar mass and stereoselectivities delivered by complexes **142**–**145**. For example, complex **145** has two initiating ethoxy groups, and thus dissociation during polymerization would be expected to double the propagation rate as two active species would be present instead of one. Yet this was not observed, and similar propagation rates were reported relative to **144** and **147**. However, it should be noted that the polymer *M*_n_ values obtained using **145** were half of those obtained with **142** and **144**, suggesting one polymer chain per ethoxide. Enantiopure (*R*,*R*/*R*,*R*)-**142** and (*R*,*R*/*R*,*R*)-**145** complexes were also synthesized and screened for *rac*-LA ROP. If the dissociative mechanism occurred, then both complexes would have formed the same active species and similar stereoselectivity tendencies would be expected. On the contrary, *P*_m_ values of 0.48 and 0.65 were determined for (*R*,*R*/*R*,*R*)-**142** and (*R*,*R*/*R*,*R*)-**145**, respectively, providing further support for the bimetallic pathway.

**Figure 24:**
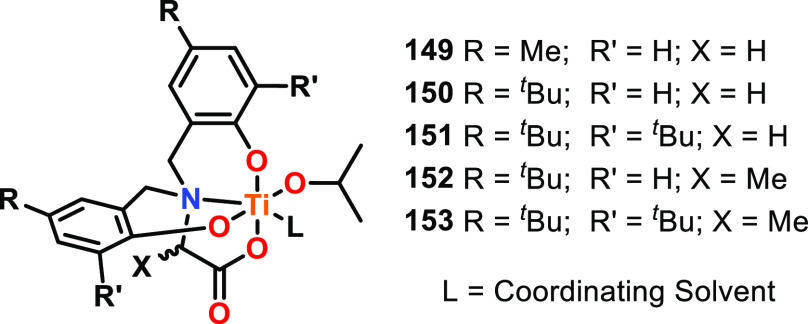
Monomeric representation of titanium complexes that form aggregates in solution and are active catalysts for *rac*-LA ROP.^111^

Breaking up aggregates into monomeric species has the potential to decrease steric congestion at the active metal centers, thus facilitating lactone coordination and insertion, and enhancing the polymerization rate.^129,62^ The aggregation state can be influenced by multiple factors, including the solvent, concentration and the steric bulk of the ligand. While increasing the ligand steric bulk often decreases the aggregation state and enhances the catalyst activity, this is not always the case.^130−132^ McIntosh and co-workers reported a series of titanium catalysts including complexes **149**–**153** for *rac*-LA ROP (Figure 24).^111^ While **149**–**153** displayed dynamic mixtures of different aggregation states in solution, DOSY studies in noncoordinating CDCl_3_ solvent showed a clear trend for complexes **149**–**151**, where increasing the steric bulk of the ligand decreased the aggregation state (**149** > **150** > **151**). This correlated to enhanced catalyst activity for *rac*-LA under solution polymerization conditions (**151** > **150** > **149**), which was attributed to lower aggregation states increasing monomer access to the active metal centers thus facilitating polymerization. Under melt conditions, complex **150** was more active than **151**, which was ascribed to *rac*-LA behaving as a strong donor solvent to break up the aggregates. Introducing a Me group into the ligand backbone increased the steric bulk, which decreased the aggregation state (**152** and **153** vs analogous **150** and **151**) yet decreased the catalyst activity in solution-state polymerizations. This was attributed to the steric bulk of the ligand disfavoring monomer and/or polymer coordination to the metal center. Overall, these studies show that the relationship between the aggregation state and catalyst activity is not necessarily straightforward.

**Figure 25:**
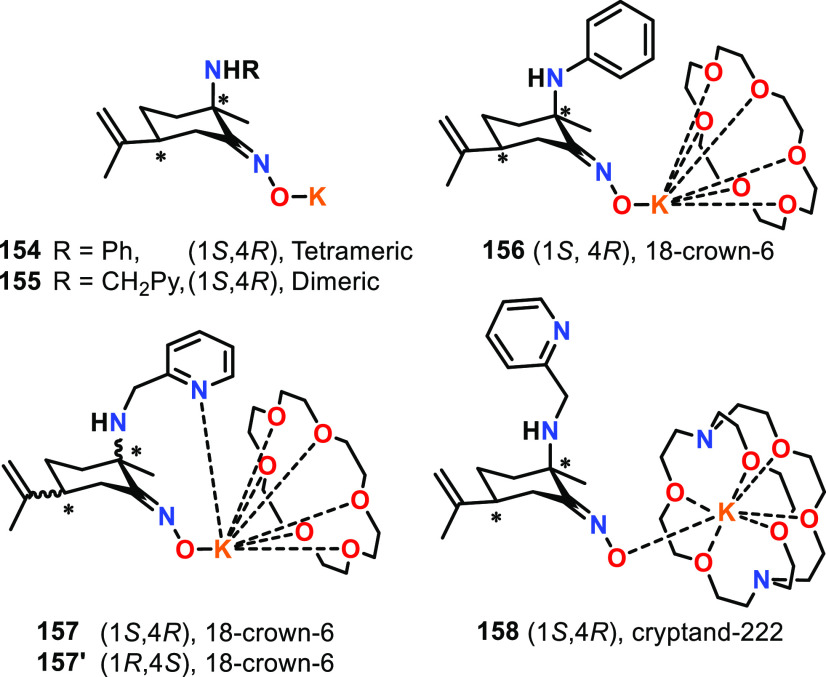
Potassium terpene-based complexes for LA ROP, which are disaggregated through the incorporation of 18-crown-6 or cryptand-222 (where py is pyridyl).^129^

As steric accessibility of the metal center is a key parameter in ROP, the use of Lewis donor ligands such as crown ethers or cryptand-222 have been investigated as a method of decreasing aggregated catalysts into monomeric species.^129^ Cano, Mosquera, and co-workers prepared chiral Li, Na, and K complexes based on a terpene-derived ligand that showed activity in *rac*-LA ROP, and the K complexes were investigated for disaggregation (Figure 25, **154**–**158**). It is worth noting that chiral complexes are of particular interest in ROP, as they can deliver stereocontrolled *rac*-LA ROP through an enantiomorphic site control mechanism,^116^ where the catalyst chirality influences whether d-LA or l-LA is preferentially inserted into the propagating polymer chain. Prior to the addition of 18-crown-6 or cryptand-222, aggregated **154** and **155** were tetrameric and dimeric in C_6_D_6_, respectively. Complex **154** gave atactic PLA (25 °C, toluene, [*rac*-LA]:[Cat] 100:1), whereas **155** displayed a slight isotactic bias (*P*_m_ = 0.6). The *M*_n_ values were higher than expected, and decreasing the reaction temperature further increased the *M*_n_ values, suggesting that not all of the catalyst was active as aggregation causes steric inaccessibility of some metal centers. Addition of cryptand-222 to complex **155** shut down the reactivity toward *rac*-LA ROP, which was attributed to the steric inaccessibility of the potassium cation in **158**. In contrast, addition of 18-crown-6 ether to **154** and **155**, to form **156** and **157**, maintained activity. For example, complex **156** gave 99% conversion to PLA after 2 min at 25 °C, giving a similar activity to tetrameric **154** (also >99% conversion) and outperforming dimeric **155** (58% conversion).

At −70 °C, complexes **154**–**156** became inactive to ROP, implying that aggregation occurs at low temperatures. In contrast, complex **157** was active for ROP and gave >99% conversion at −70 °C (toluene, [*rac*-LA]:[Cat] 100:1), indicating that 18-crown-6 ether inhibits aggregation at low temperatures. Under these conditions, **157** generated highly isotactic PLA (*P*_m_ = 0.85), which could be further increased to *P*_m_ = 0.93 by incorporating 10 equiv of BnOH. Complexes **157** and **157′** were tested for l-LA ROP, with **157** giving >99% conversion after 5 min whereas **157′** delivered 63% conversion after 120 min; this indicates the stereochemical preference of **157** for l-LA.

The aforementioned examples highlight that aggregated catalysts can effectively catalyze the ROP of LA and that some aggregates, including simple alkali and alkaline earth metal–alkyls and alkoxides, can deliver stereocontrol through a chain end control mechanism. However, the process of aggregation can sterically shield the active metal centers from lactone monomers, which can reduce the polymerization rates. Strategies to overcome this obstacle have included the use of Lewis base donors to decrease the aggregation state, either by performing LA ROP under bulk conditions or by using bulky ligands such as crown ethers. However, it is important to note that aggregation processes are often dynamic, are dependent on the reaction conditions such as temperature, solvent, and concentration, and that reduced aggregation states do not always correlate to improved catalyst performance. Overall, the steric accessibility at the metal center is a key factor.

## 3 Multimetallic Catalysts for the ROP of Cyclic Ethers

Similar to cyclic esters, the ROP of cyclic ethers (e.g., epoxides) involves the breaking and making of C–O bonds and is driven by the release of ring strain.^133^ Early epoxide ROP was performed using simple metal salts as the catalyst, such as iron(III) chloride.^134−139^ Notably, isotactic polypropylene and polypropylene oxides were invented at a similar time,^140,141^ and the first synthesis of isotactic polypropylene oxide predates that of isotactic polypropylene.^42^ However, polypropylene oxide received far less attention, which may be partially due to the relative lack of catalyst development for epoxide ROP.

**Scheme 7:**
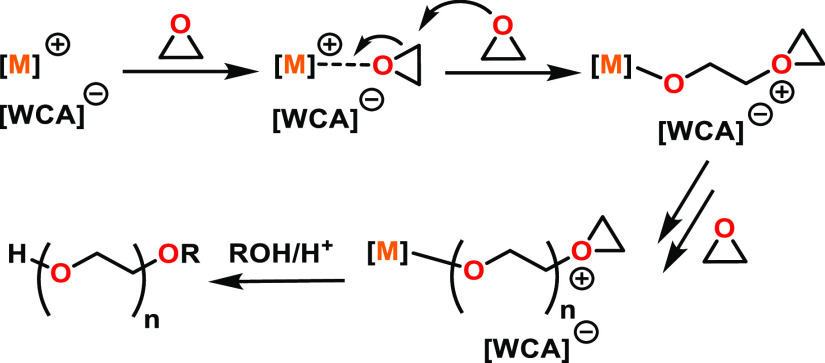
Proposed Mechanism for Cationic ROP of Epoxides, Where [M] Is a Ligand-Supported Metal Center and [WCA]^−^ Is a Weakly Coordinating Anion^142^

Epoxides are typically polymerized through three main routes: (i) cationic, (ii) anionic, and (iii) coordination–insertion mechanisms. To achieve well-controlled polymerization, the active propagation site should generally be positioned near the metal center. In cationic mechanisms, the positive charge progresses along the active polymer chain end until termination occurs (Scheme 7). As the metal centers are far from the active propagation site, they play a little or no role except from the initiation. Multimetallic cooperativity is therefore less relevant in the cationic ROP of epoxides than for other epoxide polymerization mechanisms,^142^ such as the CIM, which can deliver polyethers with controlled *M*_n_, dispersity, stereoselectivity, enantioselectivity, regioregularity, and crystallinity.^42^ While there are indications that multimetallic catalysts can display improved performance in both cyclic ester and cyclic ether ROP, multimetallic catalysts remain underexplored in the latter.^143,144^

**Figure 26:**
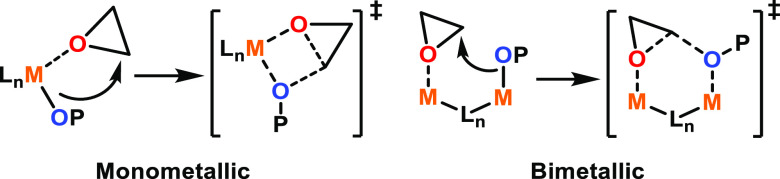
Proposed transition states for monometallic and bimetallic ROP of cyclic ethers.^42^

**Scheme 8:**
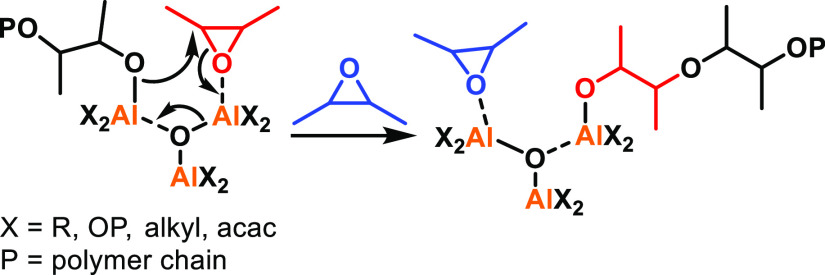
Proposed Chain Shuttling Mechanism for ROP of Cyclic Ethers by Metal Aggregates^147^

In the 20th century, several multimetallic aggregates were reported for the enantioselective ROP of epoxides, including both homo- and heterometallic systems, which were mainly based on Zn or Al. These aggregates were proposed to operate through a CIM and generated crystalline/semicrystalline polymers.^145−147^ To the best of our knowledge, there is limited information reported on a conclusive mechanism for epoxide ROP catalyzed by metal aggregates, yet bimetallic transition states have been proposed to be less strained and more favorable than monometallic transition states (Figure 26).^42^ The CIM was proposed to involve monomer coordination to one of the metal centers, followed by alkoxide transfer from the other metal to ring open the epoxide, with propagation occurring through a chain shuttling mechanism (Scheme 8).^148^

Yuan, Yao, and co-workers reported a comparative study of mono- and bimetallic aluminum catalysts for the ROP of PO and cyclohexene oxide (CHO), where the bimetallic catalysts outperformed their monometallic congeners (Figure 2, monometallic complex **9** and bimetallic **9.AlMe**_**3**_, which is a Lewis adduct of **9** with AlMe_3_ coordinated to the phenolic O). Kinetic studies showed the polymerization rate of **9.AlMe**_**3**_ to be four times higher than that of **9** (hexane, 30 °C, [CHO]:[Al] 1000:1).^149^ MALDI-ToF end-group analysis of the poly(cyclohexene oxide) oligomers showed methyl-capped polymers, providing evidence for the CIM pathway. Catalysts **9** and **9.AlMe**_**3**_ displayed a similar reactivity trend with the more challenging ROP of PO, albeit with a reduced polymerization rate, as **9** gave 7% yield in 12 h whereas **9.AlMe**_**3**_ gave 84% yield in 12 h (80 °C, neat PO, with a [PO]:[Cat] loading of 200:2 for monometallic **9**, and 100:1 for bimetallic **9.AlMe**_**3**_).

**Scheme 9:**
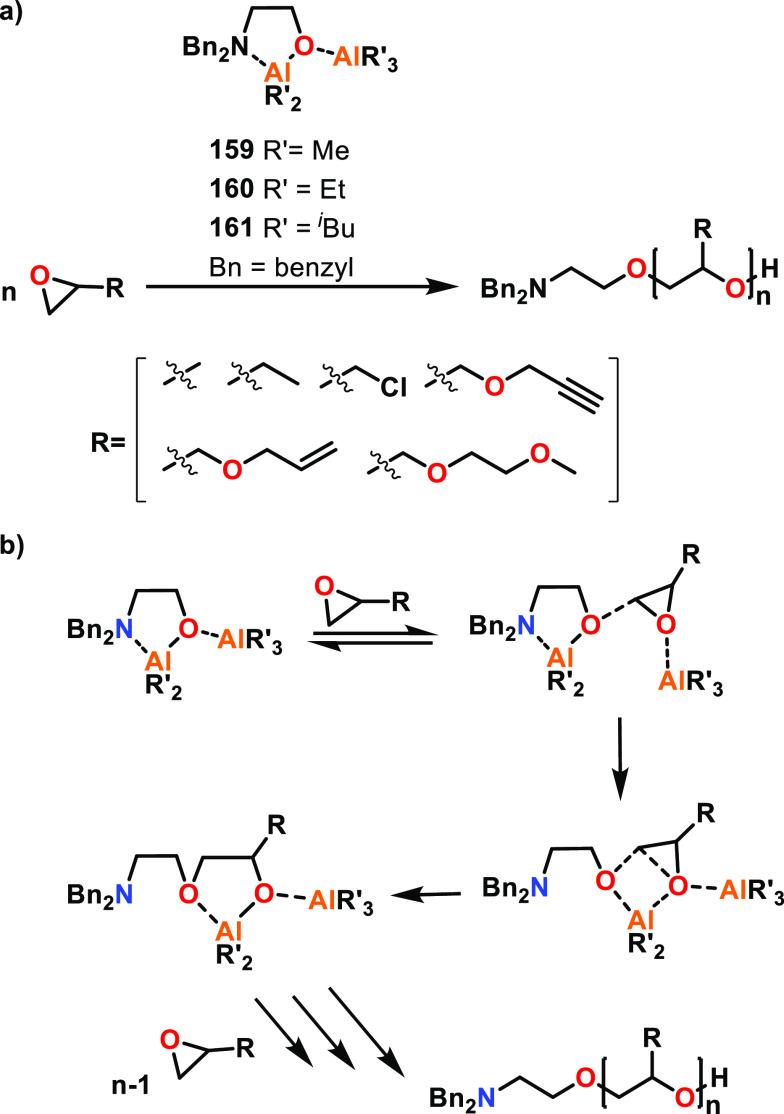
(a) ROP of Epoxides by Alkyl Aluminum Dimers. (b) Proposed Epoxide ROP Mechanism for **159**–**161**^150,151^

In 2017, Lynd and co-workers reported a series of organoaluminum complexes (**159**–**161**) for the ROP of a broad range of asymmetric epoxides, giving conversions of up to 99% (Scheme 9).^150,151^ The ligands played a key role in the catalyst performance. For example, the ^*i*^Bu analogue **161** was four times more active than the Et analogue **160** for the ROP of allyl glycidyl ether (*k*_p_ = 1.10 × 10^–3^ M^–1^ s^–1^ vs 0.27 × 10^–3^ M^–1^ s^–1^, respectively). XRD studies showed that **161** has a longer Al–O dative bond (1.92 Å) than **159** and **160** (1.90 and 1.89 Å, respectively), facilitating Al–O bond cleavage in **161**; this was proposed to assist monomer coordination and ring-opening, thus increasing the propagation rate. This mechanism was supported by the presence of ligand end groups in the polymer chains (Scheme 9b), as observed by ^1^H NMR spectroscopy.^151^

**Figure 27:**
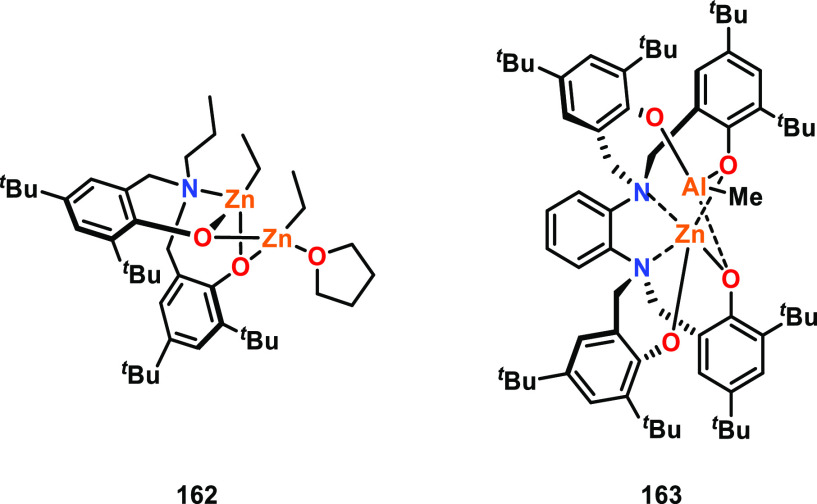
Homo- and heterobimetallic zinc and aluminum compounds for the ROP of epoxides. Heterometallic **163** displayed high activities for CHO ROP (TOF values up to 24 600 h^–1^).^152,154^

The bimetallic zinc catalyst **162** has also been reported for epoxide ROP (Figure 27), giving a TOF of 65 h^–1^ and polymers with *M*_n_ = 17.8 kg mol^–1^ and *Đ* = 1.9 (20 °C, [CHO]:[Cat]:[BnOH] 200:1:2).^152^ Benzoxide end groups were observed by ^1^H NMR spectroscopy, suggesting that the polymerization was initiated by Zn-benzoxide. While active, it is worth noting that bimetallic **162** displays lower activities than some monometallic zinc catalysts.^153^

Recently, a heterobimetallic Al–Zn complex **163** (Figure 27) was reported for the ROP of CHO, which achieved TOF values of up to 24 600 h^–1^ under solvent-free conditions (30 °C, [CHO]:[Cat] 1000:1).^154^ In contrast, the respective monometallic compounds were inactive due to coordinative saturation at the metal centers. While the exact mechanism remains unclear, kinetic studies performed in toluene solvent at 30 °C revealed a first-order dependency on both the monomer and the catalyst concentration.

**Figure 28:**
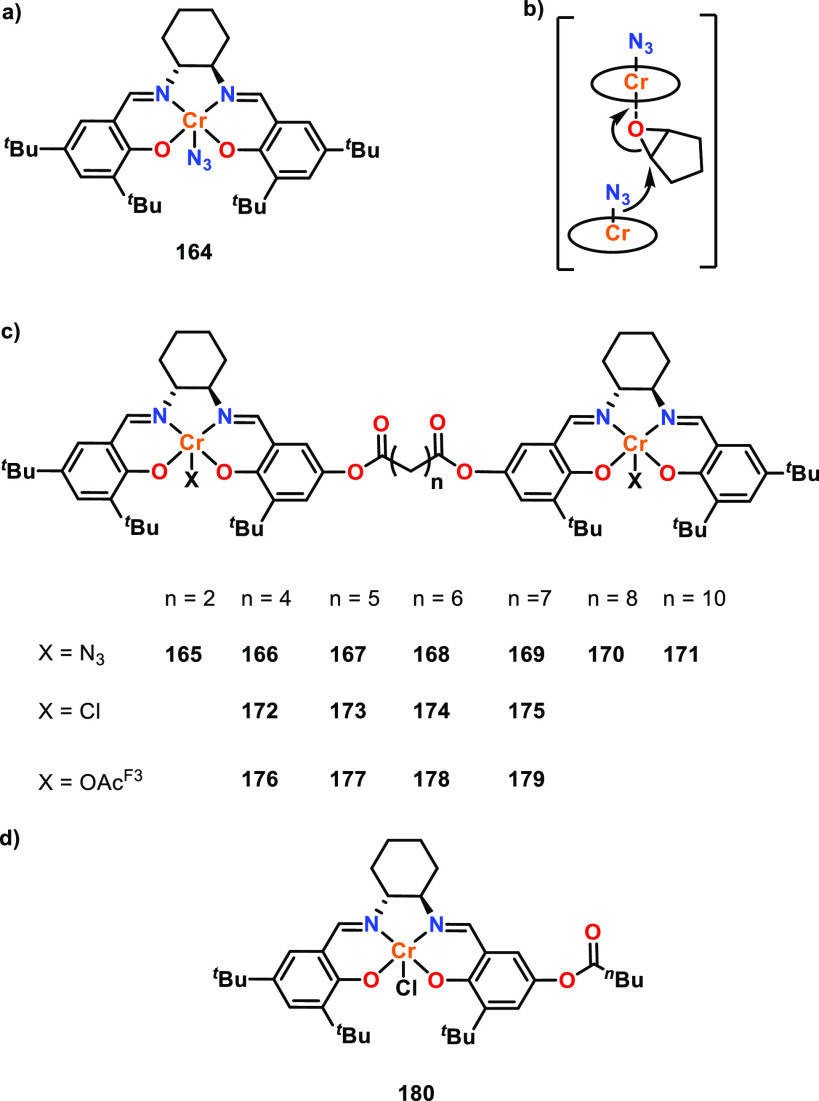
(a) Monometallic (salen)Cr(III) catalyst. (b) Proposed head to tail arrangement in (salen)Cr(III) epoxide ring-opening catalysts. (c) Covalently tethered bimetallic (salen)Cr(III) catalysts **165**–**179**. (d) Monometallic (salen)Cr(III) analogue of **172**–**175**. For azide complexes **165**–**171**, **167** (*n* = 5) was the most active for CPO ring-opening; for the chloride (**172**–**175**) and trifluoroacetate (**176**–**179**), the most active were **174** and **178**, respectively, for PO ROP (*n* = 6 for both).^155−157^

In addition to the aforementioned main group complexes, the Coates group has developed multimetallic transition metal-based catalysts for epoxide ROP.^144,145^ This work was inspired by studies from Jacobsen and co-workers, which investigated the asymmetric ring-opening of epoxides rather than epoxide polymerization, and used a (salen)Cr(III) (**164**)/trimethylsilyl azide (TMS-N_3_) catalyst system (Figure 28a). Kinetic studies showed the reaction was second order in catalyst, suggesting simultaneous activation of the azide group and epoxide in a bimetallic rate-determining step (Figure 28b).^155^ A series of covalently linked bimetallic (salen)Cr(III) catalysts (**165**–**171**, Figure 28c) were subsequently developed for the asymmetric ring-opening of cyclopentene oxide (CPO) using TMS-N_3_ or HN_3_ as the azide source. The highest rate was obtained with compound **167**, suggesting that the linking unit *n* = 5 provided an optimum distance for the two metal centers to act cooperatively.^155,156^ This study provided key insights for epoxide ROP, as the cooperativity was attributed to the need for a “head to tail” arrangement of the two metals, with the Cr–N_3_ initiating unit of one metal situated close to the other, Cr-epoxide unit.

Coates and co-workers subsequently developed bimetallic (salen)Cr(III) catalysts for epoxide ROP that, in the presence of [PPN]Cl/[PPN][OAc^F3^], delivered isotactic semicrystalline polypropylene oxide (**172**–**179**, Figure 28c).^157^ By varying the number of CH_2_ groups from n = 4–7 (**172**–**179**), the Cr–Cr distance was tuned for optimum metal–metal cooperativity. The highest TOF values of 627 and 640 h^–1^ were observed with **174** and **178**, suggesting that *n* = 6 provides an appropriate M–M distance for metal–metal cooperativity in PO ROP. Excellent enantioselectivity and molar mass control was observed by using diols such as 1,6-hexanediol as a chain transfer agent. In contrast, the corresponding monometallic (salen)Cr(III) compound, **180** (Figure 28d), was essentially inactive for PO ROP under the conditions tested, emphasizing the opportunity for multimetallic cooperativity in bimetallic (salen)Cr(III) catalysts.^157^

**Scheme 10:**
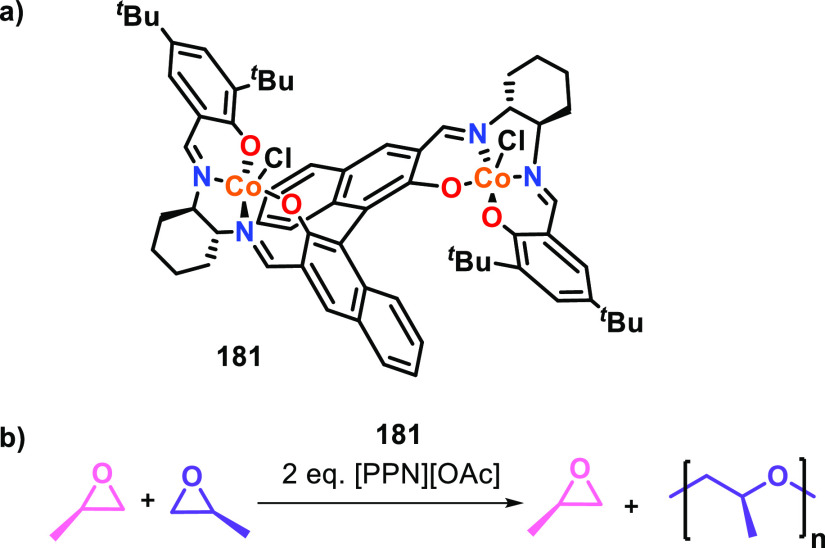
(a) Bimetallic Cobalt Catalyst **181**. (b) Enantioselective Epoxide ROP^158^

Highly active and enantioselective bimetallic cobalt catalysts have been reported for epoxide ROP (ee >99%), based on dinucleating salen ligand scaffolds (Scheme 10).^158^ The two metal binding pockets are coordinatively connected by a chiral binaphthol linkage. The oxidized, Co^II^ analogue of **181** was characterized by XRD studies and displayed a Co–Co distance of 5.96 Å (a second, ethanol-bound molecule was also present in the unit cell with a Co–Co distance of 5.22 Å).^158^ The former value is close to 6 Å, which was proposed as an optimum M–M distance in epoxide ROP studies using bimetallic chromium catalysts.^157^ Following success with some enantiopure monosubstituted epoxides, a series of racemic monosubstituted epoxides was tested. Using complex **181** along with cocatalyst [PPN][OAc] as an external initiator gave enantioselective polymerization of racemic epoxides with a conversion of up to 34% and a selectivity factor (for the ‘*S*’ isomer) of up to 370 (0 °C, toluene, [PO]:[Cat]:[PPN][OAc] 4000:1:2). The racemic analogue of catalyst **181** was also reported and generated highly isotactic polyethers from racemic epoxides with >99% conversion for all of the epoxides investigated.^159^

While relatively few multimetallic catalysts have been reported for epoxide ROP, most are from the p-and d-block, with early examples based on aggregated aluminum and zinc-alkoxides and more recent reports focusing on ligand supported Cr and Co complexes. Multimetallic cooperativity has been reported for catalysts operating through a coordination insertion pathway, rather than those following a cationic mechanism where the active polymer chain end is far removed from the coordination sphere and influence of the metals. While several heterometallic catalysts have been reported for epoxide/CO_2_ and epoxide/anhydride ring-opening copolymerizations, discrete heterometallic catalysts for epoxide ROP currently remain scarce.

## 4 Summary and Outlook

Overall, these studies show that multimetallic cooperativity can be exploited to deliver superior catalyst performance in the ROP of cyclic esters and ethers compared to the monometallic analogues. Emerging trends highlight that the metal–metal (M–M) proximity, the ligand conformation, flexibility and sterics, and the electronic nature of the metal centers are all key factors influencing multimetallic cooperativity.

The M–M distance is pivotal, as if it is “too long” the metal centers can act separately, yet if the distance is “too short” this can prevent effective polymerization due to steric hindrance. While XRD studies are not reported for all of the multimetallic catalysts for cyclic esters described here, the M–M distance generally ranges from 2.9 to 8.0 Å. There is no clear trend in terms of the optimal M–M distance, which appears to change for different catalyst systems and may be metal dependent. For example, some Al catalysts have been reported where a M–M proximity of 5 Å is better than 3 Å, and several studies have indicated that short Al–Al distances can hamper the catalyst activity. In contrast, some Zn catalysts have shown the opposite trend, with a M–M distance of 3 Å outperforming those with a 5 Å separation. This may be linked to differences in the polymerization mechanism. While most multimetallic catalysts have been proposed to proceed via coordination insertion mechanisms, M–M proximity can enable mechanisms where two metals can simultaneously coordinate a monomer or a bridging propagating chain (proposed for some dizinc catalysts) or can facilitate a chain-shuttling mechanism (proposed for some dialuminum catalysts, refer to section 2.1.4 for details). However, it is important to note that more information is required to understand the overarching mechanistic differences between diverse multimetallic catalysts. It would therefore benefit the field if future research included detailed mechanistic studies and identification of metal–metal proximity for a range of multimetallic catalysts, including catalysts based on metals from across the periodic table.

The ligand flexibility and conformation, together with the metal geometry, can be important in dictating the M–M proximity and/or delivering sterically accessible metal centers for monomer coordination. Steric availability appears to be key across the different classes of multimetallic systems reviewed. For example, catalysts with a “folded” conformation can open up monomer coordination sites compared to their “open” analogues, leading to enhanced activities.^58^ Some trimetallic aluminum catalysts featuring a central square pyramidal aluminum have shown enhanced activities compared to their bimetallic or tetrametallic analogues, with monomer coordination proposed to occur at the central Al center.^65,123,124^ The steric availability can also influence the polymerization mechanism. For example, some studies suggest that steric accessibility determines whether both metals act cooperatively (e.g., with one coordinating the monomer and the other providing the nucleophile) or whether polymerization occurs simply at one metal center.^34^ However, steric accessibility is not always straightforward to predict. For example, sterically bulky ligands can cause congestion at key catalytic sites, thus blocking monomer access and slowing catalyst activity. Yet removing steric bulk from the ligand can cause catalyst aggregation, which can also hamper monomer access to the metal centers. Overall, there is a subtle balance in designing ligands to prevent aggregation while maintaining steric accessibility at the metal centers.

The electronic environment of a metal center can be modulated by the ligand and/or the presence of a second metal. Cooperative multimetallic catalysts have been reported where two or more metal centers are in electronic communication, either through a bridging heteroatom (typically a phenoxide-O) or a conjugated ligand backbone. This electronic communication often leads to synergistic effects, especially in trimetallic and heterometallic complexes. In the latter, this electronic communication can lead to the formation of “ate” complexes, which means that each metal can be tailored for a specific key step of ROP: monomer activation or nucleophilic attack. Expanding the library of heterocombinations in ROP catalyst design opens new possibilities for multimetallic catalysts. While most cooperative multimetallic catalysts have delivered enhanced polymerization rates, some have also delivered enhanced tacticity control in lactide ROP, particularly with aggregate-based systems.

Overall, the key factors of M–M proximity and metal accessibility, ligand flexibility and conformation, and the electronic environment of the metals are often closely interlinked. Each of these factors may influence the polymerization mechanism, and thus understanding the true origins of cooperativity can be challenging. Direct comparison between the multimetallic complexes and the monometallic analogues is not always possible, as this can introduce differences in the metal coordination environments and the metal concentration. Where possible, benchmarking against directly comparable species, combined with analysis of the solution-state structures, is beneficial for identifying whether or not catalysts are truly cooperative. At present, there is no consensus on a multimetallic ROP mechanism for cyclic esters, and indeed, different catalysts are likely to follow different mechanisms. Identifying the multimetallic mechanisms for a broad range of cyclic ester ROP catalysts would help to build understanding of mechanistic trends for different metals, enabling targeted catalyst design.

Multimetallic complexes display some similarities to monometallic complexes, e.g., the ligand electronics and flexibility influence the catalyst performance in both. However, common trends observed for monometallic catalysts are not always mirrored with multimetallic catalysis. For example, some heterometallic Al-salen catalysts have been reported where the more rigid catalysts displayed the highest activity; the opposite trend is generally reported for monometallic Al-salen complexes.

In comparison to cyclic esters, the ROP of cyclic ethers is relatively underexplored, yet M–M proximity can be advantageous. Furthermore, there are some synergies between multimetallic catalyst design for cyclic ester and cyclic ether ROP. Therefore, understanding the origins of multimetallic cooperativity within these systems may help to provide a synthetic shortcut to improve catalyst design and translate knowledge to other polymerization processes and other fields of chemistry in the future.
